# Interferon alpha inducible protein 6 is a negative regulator of innate immune responses by modulating RIG-I activation

**DOI:** 10.3389/fimmu.2023.1105309

**Published:** 2023-01-30

**Authors:** Laura Villamayor, Vanessa Rivero, Darío López-García, David J. Topham, Luis Martínez-Sobrido, Aitor Nogales, Marta L. DeDiego

**Affiliations:** ^1^ Department of Molecular and Cell Biology. Centro Nacional de Biotecnología (CNB-CSIC), Madrid, Spain; ^2^ David H. Smith Center for Vaccine Biology and Immunology, Department of Microbiology and Immunology, University of Rochester Medical Center, Rochester, NY, United States; ^3^ Disease Intervention and Prevention and Population Health Programs, Texas Biomedical Research Institute, San Antonio, TX, United States; ^4^ Center for Animal Health Research, CISA-INIA-CSIC, Valdeolmos, Madrid, Spain

**Keywords:** IFI6, interferon, innate immunity, RIG-I, RNA binding, inflammation, influenza virus, coronavirus

## Abstract

Interferons (IFNs), IFN-stimulated genes (ISGs), and inflammatory cytokines mediate innate immune responses, and are essential to establish an antiviral response. Within the innate immune responses, retinoic acid-inducible gene I (RIG-I) is a key sensor of virus infections, mediating the transcriptional induction of IFNs and inflammatory proteins. Nevertheless, since excessive responses could be detrimental to the host, these responses need to be tightly regulated. In this work, we describe, for the first time, how knocking-down or knocking-out the expression of IFN alpha-inducible protein 6 (IFI6) increases IFN, ISG, and pro-inflammatory cytokine expression after the infections with Influenza A Virus (IAV), Severe Acute Respiratory Syndrome Coronavirus 2 (SARS-CoV-2), and Sendai Virus (SeV), or poly(I:C) transfection. We also show how overexpression of IFI6 produces the opposite effect, *in vitro* and *in vivo*, indicating that IFI6 negatively modulates the induction of innate immune responses. Knocking-out or knocking-down the expression of IFI6 diminishes the production of infectious IAV and SARS-CoV-2, most likely because of its effect on antiviral responses. Importantly, we report a novel interaction of IFI6 with RIG-I, most likely mediated through binding to RNA, that affects RIG-I activation, providing a molecular mechanism for the effect of IFI6 on negatively regulating innate immunity. Remarkably, these new functions of IFI6 could be targeted to treat diseases associated with an exacerbated induction of innate immune responses and to combat viral infections, such as IAV and SARS-CoV-2.

## Introduction

Innate immune responses mediated by type I and III interferons (IFNs), IFN-stimulated genes (ISGs) and pro-inflammatory cytokines, are the first line of defence against the infections caused by viruses, since these host proteins restrict viral replication by different mechanisms (1, 2). Some pathogen molecules, such as glycoproteins, proteoglycans and nucleic acids motifs, such as double-stranded (ds)RNA and 5´phosphate single-stranded (ss)RNA motifs, are known as Pathogen-associated Molecular Patterns (PAMPs), and are recognized by specialized receptors, known as pattern recognition receptors (PRRs) (3). The recognition of PAMPs by PRRs, such as Toll-like receptor 3 (TLR-3), melanoma differentiation-associated protein 5 (MDA-5) and retinoic acid-inducible gene I (RIG-I), initiate converging signalling pathways leading to the activation of transcription factors such as IFN regulatory factors 3 and 7 (IRF-3 and IRF-7, respectively), nuclear factor kB (NF-kB) and activating protein 1 (AP-1), and the subsequent production of type I and type III IFNs, and inflammatory cytokines (4). IFN molecules bind to their cell surface receptors comprised of dimers of IFNAR1 and IFNAR2 proteins, for type I IFNs, and dimers of IFNLR1 and IL10RB for type III IFNs. This binding initiates a signalling cascade through Janus kinase signal transducer and activator of transcription (JAK-STAT) pathway (2). This cascade leads to the transcription of many ISGs encoding antiviral activities mediated by different molecular mechanisms (5). In addition to the classical ISGs that limit viral production, there are other ISGs that play a negative regulatory role, aimed at preventing an excessive response and inflammation, which can be deleterious to the host (6–9) or aimed at playing a positive regulatory roles in the induction of innate immune responses (8, 9). In this regard, we have previously described that ISGs IFI44 and IFI44L negatively modulate IFN responses and augment virus replication, as well as the novel molecular mechanisms mediating their effect on modulating the innate immune responses (10, 11).

RIG-I is one of the main PRRs, which primarily recognizes short double-stranded (ds)RNAs and single-stranded (ss)RNAs with 5′ triphosphate groups (12, 13). RIG-I consists of two caspase activating and recruiting domains (CARD) at the N-terminus, which mediate downstream signal transduction, followed by a flexible hinge region, a central helicase domain, another flexible hinge region, and a carboxy-terminal domain (CTD) that recognizes and binds RNAs (13).

Despite all the knowledge we have in this field, there are still many ISGs whose function is still unknown or only partially understood. IFN alpha inducible protein 6 (IFI6), also known as G1P3, ISG16, and IFI6-16, is an ISG that belongs to the FAM14 family, composed by four genes in humans (IFI6, IFI27, IFI27L1 and IFI27L2) and three in mice (ISG12a, ISG12b1 and ISG12b2) (14). IFI6 is a 13-kDa hydrophobic protein of 130 amino acids (14), which has putative transmembrane helices (15). IFI6 was considered to be a mitochondrial protein that plays a critical role in immunomodulation, and it has an antiapoptotic function blocking the mitochondrial release of cytochrome c (16) and stabilizing mitochondrial membrane potential in Dengue virus (DENV)-infected cells (17). Nevertheless, more recent studies have shown that IFI6 is mainly localized at the endoplasmic reticulum (15, 18). IFI6 roles in innate immune responses as an ISG are still unknown, but as it is upregulated by type I IFN, there is a great interest in delving into its importance in antimicrobial cellular pathways. Several studies have assessed IFI6 role against viral infections in order to describe possible mechanisms of action. IFI6 inhibits replication and gene expression of hepatitis B virus (HBV) in cell cultures and in a murine model (19). Furthermore, this inhibitory effect on HBV expression is due to the binding of IFI6 to the EnhII/Cp promoter of the virus (19). Similarly, IFI6 has been described as an inhibitor of hepatitis C virus (HCV) entry and replication, and this effect is due to IFI6´s ability to impair CD81 and claudin 1 (CLDN1) interactions by inhibiting the function of the epidermal growth factor receptor (EGFR) kinase (20). The binding of the flavivirus HCV to human hepatocytes induces EGFR activation, which is an important cofactor for the entry process of multiple viruses, including HCV (21). Thus, inhibiting EGFR kinase activity diminished infection of all major HCV genotypes in cultured cells and in human liver chimeric mouse model (21). In addition to HCV, several studies have demonstrated the antiviral effect of IFI6 upon infection with other flaviviruses such as DENV, West Nile Virus (WNV) or Yellow Fever Virus (YFV) (15, 22–24). IFI6 interacts with the chaperone BiP at the endoplasmic reticulum (ER) membrane, thus inhibiting flavivirus replication by preventing the formation of virus-induced ER membrane invaginations that protrudes inwards the ER, which are double-membrane vesicles that flaviviruses use for their replication (15). Therefore, despite IFI6 has recently proven to be important in innate immune response against viruses, its biological and immunomodulatory mechanisms are yet to be completely elucidated.

In this work we describe, for the first time, that IFI6 positively affects infection of two highly relevant human respiratory RNA viruses (IAV and SARS-CoV-2). In addition, we find a completely novel function for IFI6 in negatively regulating innate immune responses induced after viral infections in cell cultures and *in vivo*. The molecular mechanism involves the interaction of IFI6 with RNAs and RIG-I, negatively modulating RIG-I activation, and therefore, innate immune responses.

## Materials and methods

### Cells

Madin-Darby Canine Kidney epithelial (MDCK, American Type Culture collection, ATCC CCL-34), human embryonic kidney (293T; ATCC CRL-11268), human lung epithelial carcinoma (A549; ATCC CCL-185), and African green monkey kidney epithelial E6 (Vero E6, kindly provided by Prof. Luis Enjuanes, Centro Nacional de Biotecnología, CNB-CSIC, Spain) cells were grown at 37°C in air enriched with 5% CO_2_ using Dulbecco’s modified Eagle’s medium (DMEM, Gibco) supplemented with 10% fetal bovine serum (Gibco), and 50 μg/ml gentamicin (Gibco). A549 cells overexpressing human ACE-2 (hACE-2, A549-ACE-2), generated in our laboratory, were grown in the same media containing 2.5 μg/ml of blasticidin (ThermoFisher Scientific). Human HAP-1 near-haploid wild-type (WT) cells, derived from a human leukemia, and HAP-1 cells knock-out (KO) for IFI6 using the CRISPR/Cas9 technology were obtained from Horizon Discovery, Inc. These cells were grown at 37°C in air enriched with 5% CO_2_ using Iscove´s Modified Dulbecco´s medium supplemented with 10% fetal bovine serum (Gibco), and 50 μg/ml gentamicin (Gibco). The non-tumorigenic human bronchial epithelial cell line BEAS-2B (CRL-9609), was obtained from the ATCC and grown at 37°C in air enriched with 5% CO using RPMI medium (Gibco) supplemented with 10% fetal bovine serum (Gibco), and 50 μg/ml gentamicin (Gibco).

### Viruses

Virus stocks of IAV A/Puerto Rico/8/1934 H1N1 (PR8) were grown in MDCK cells (25) under BSL2 conditions. Sendai virus (SeV), Cantell strain (26), was grown in embryonated chicken eggs under BSL2 conditions. The recombinant Vesicular Stomatitis Virus, Indiana strain, encoding the green fluorescent protein (GFP) (rVSV-GFP) (27), and the SARS-CoV-2 were grown in Vero E6 cells, under BSL2 and BSL3 conditions, respectively. SARS-CoV-2, isolated in Vero E6 cells, was originating from a nasal swab from a patient infected in Madrid, Spain, at the beginning of 2020, and was kindly provided by prof. Luis Enjuanes, at Centro Nacional de Biotecnología, CNB-CSIC, Spain (unpublished results).

### Virus titrations

Parental and recombinant IAVs encoding IFI6 and mCherry (IAV-IFI6 and IAV-mCherry, respectively) were titrated in confluent MDCK cells seeded in 96-well plates by immunofocus assay (fluorescent focus units, FFU/ml), as previously described (28). For IAV infections 1 μg/ml of tosylsulfonyl phenylalanyl chloromethyl ketone (TPCK)-treated trypsin (Sigma) was added to the media. rVSV-GFP was titrated by plaque assay (plaque forming units, PFU/ml) in confluent Vero E6 cells seeded in 24-well plates, as previously described (29). SARS-CoV-2 was titrated by plaque assay (PFU/ml) in confluent Vero E6 cells seeded in 24-well plates, as previously described (30).

### Plasmids

Polymerase II expression pCAGGS plasmids encoding IFI6 (GenBank accession number NM_022873) C-terminally fused to an HA epitope tag (pCAGGS-IFI6-HA), and PRKRA (Protein Activator of Interferon Induced Protein Kinase EIF2AK2) fused to a FLAG epitope tag (GenBank accession number NM_003690.5) were generated by RT-PCR using total RNA isolated from human epithelial A549 cells and cloned using standard techniques (primers available upon request). pCAGGS plasmids expressing RIG-I protein (GenBank accession number AF038963.1) fused to a FLAG epitope tag (pCAGGS-RIG-I-FLAG), and GFP, were previously described (31). pMP31 plasmid encoding mitochondrial antiviral signaling protein (MAVS) fused to a FLAG epitope (pMP31-MAVS-FLAG) was obtained from Addgene, and previously described (32).

To generate IFI6 KO cells, the best short guide RNAs (sgRNAs) to be used, were selected using the webpages (https://www.atum.bio/eCommerce/cas9/input?multipleContacts=false and http://crispor.tefor.net). The sgRNA sequences selected were: 5´-GCTGACCTTCATGGCCGTCGG-3´and 5´-GCCCTGACCTTCATGGCCGT-3´. The cDNAs complementary to the two different sgRNAs were cloned in the pX330 plasmid which was modified to encode a puromycin-resistance gene (kindly provided by Dr. Pedro A. Mateos, Universidad de Alcalá de Henares, Spain), expressing the RNA guides under the U6 promoter and encoding the Caspase 9 gene and a gene encoding for resistance to puromycin. To this end, a pair of forward and reverse oligonucleotides for the generation of each sgRNA (synthesized by IDT) were annealed and phosphorylated by incubating the forward and reverse primers with T4 polynucleotide kinase (New England Biolabs), during 30 min at 37°C, followed by 95°C during 5 min and then ramp down to 25°C, at 5°C/min. The phosphorylated and annealed primers were inserted into plasmid vector pX330 between BbsI restriction sites.

In order to generate a plasmid encoding two different 2A autoproteolytic cleavage sites and the IAV non-structural 1 (NS1) and nuclear export protein (NEP) genes expressed as independent ORFs, we used the previously described pDZ-NS-2xBsmBI plasmid (33), which contains the NS1 ORF, without the stop codon or splice acceptor site, and two BsmBI sites followed by the porcine teschovirus-1 (PTV-1) 2A autoproteolytic cleavage site and NEP (33). Then, we carried out an inverse PCR using the primers: 5´-AATTACGCGTGGA GAGGGCAGAGGAAGTCTGCTAACATGCGGTGACGTCGAGGAGAATCCTGGACCTGGGTCCGGCTGAGACGAGATCTC-3´ and 5´-AATTACGCGTTCC aacttcgcttctaattgttcccgccatttctcg-3´ to introduce the Thosea asigna virus (TAV) 2A autoproteolytic cleavage site (EGRGSLLTCGDVEENPGP). The final plasmid, named as pDZ-NSsplit2xBsmBI-2A, has the following elements: 5′-non-coding region (NCR)/NS1/link (GTRG)/TAV-2A/GSG-BsmBI/BglII/BsmBI-GSG/PTV-2A/NEP/3′- NCR. For generating recombinant IAV-IFI6 and IAV-mCherry, the plasmids pDZ-NSsplit-2xBsmBI-2A-IFI6 and pDZ-NSsplit-2xBsmBI-2A-mCherry were generated. To this end, IFI6 C-terminally fused to an HA tag and mCherry were amplified by PCR using specific primers flanked by BsmBI restriction sites, using as templates pCAGGS-IFI6-HA and pCAGGS-mCherry, and the PCR products were cloned in the pDZ-NSsplit2xBsmBI-2A plasmid.

### Virus rescue

Co-cultures (1:1) of 293T and MDCK cells were co-transfected with 1 μg of the seven ambisense WT pHW-PB2, -PB1, -PA, -HA, -NP, -NA and -M IAV-PR8 plasmids plus the pDZ-NSsplit-2xBsmBI-2A-IFI6 or pDZ-NSsplit-2xBsmBI-2A-mCherry plasmids, in 6-well plates, using lipofectamine 3000 (ThermoFisher Scientific). At 16 hours post-transfection (hpt), medium was replaced with DMEM containing antibiotics, 0.3% BSA, and 1 μg/ml of TPCK-treated trypsin (Sigma). At 48 h, cell culture supernatants were collected and used to infect fresh confluent monolayers of MDCK cells. At 3 days post-infection (dpi), recombinant viruses were plaque purified and a stock was generated by infecting MDCK cells at low MOI (0.001). Viral stocks were titrated by immunofocus assay in MDCK cells, as previously described (28). The identity of the recombinant viruses was confirmed by Sanger sequencing (Macrogen).

### Generation of A549 IFI6 KO cells

To generate A549 IFI6 KO cells, A549 cells (12-well plate format) were transfected with the pX330 plasmids (1,250 ng/well) expressing the sgRNAs, using lipofectamine 3000. At 24 hpt, cells were treated with 1 μg/ml of puromycin (InVivoGen) to select for plasmid-transfected cells. At 48 h after puromycin treatment, media was exchanged with fresh media without puromycin. Surviving cells were detached with trypsin and cloned three times by limiting dilution. Different clones were genotyped by Sanger sequencing (Macrogen).

### Overexpression of hACE-2 in A549 cells

To generate A549 cells susceptible to SARS-CoV-2 infection, parental and IFI6 KO A549 cells (6-well plate format) were transduced with a retrovirus expressing the hACE-2 protein and a gene conferring resistance to the antibiotic blasticidin (kindly provided by Dr. Pablo Gastaminza, CNB-CSIC).

### siRNA-mediated silencing

Human A549, 293T or HAP-1 cells (24 or 96-well plate format) were transfected independently with two different “silencer select” small interfering RNAs (siRNAs) specific for human IFI6 (ThermoFisher Scientific, s5441 and s5442), or with the non-targeting (NT) negative control siRNA (ThermoFisher Scientific, AM4635). All siRNAs were transfected, using lipofectamine RNAiMax (ThermoFisher Scientific), at a final concentration of 20 nM, according to the manufacturer’s instructions.

### IFN response assays

To evaluate the effect of IFI6 on IFN responses, human A549, 293T, BEAS-2B, and HAP-1 cells (24-well plate format) were transfected with siRNAs specific for IFI6, or the NT control siRNA, for 24 h. Alternatively, A549 or HAP-1 cells specifically knocked-out for IFI6 and their parental cells were seeded. Then, A549 cells were infected with IAV (multiplicity of infection, MOI 3), A549-hACE-2 cells were infected with SARS-CoV-2 (MOI 1), or BEAS-2B and 293T cells were infected with SeV (MOI 3) for 24, and/or 48 h. IAV and SARS-CoV-2 titers were determined as described above. Furthermore, cells were transfected with 60 (A549 cells); or 750; 1,500 and 3,000 (HAP-1 cells) ng/ml of polyinosinic:polycytidylic acid (poly(I:C), Sigma), using polyethylenimine (PEI, Polysciences) for 16 h. Total RNAs were extracted, and RT-qPCRs were performed, as described below. At 16 h after poly(I:C) transfection, cells were infected with rVSV-GFP (27) for 24 h. Viral titers in cell culture supernatants were determined in Vero E6 cells as previously described (29).

### RT-qPCR

mRNA levels of IFI6, IFNL1, and IFN-induced protein with tetratricopeptide repeats 2 (IFIT2) in human A549, BEAS-2B, 293T, and HAP-1 cells were analyzed using RNA extracted from cellular extracts (using total RNA extraction kit, Omega Biotek). Retrotranscriptase (RT) reactions were performed using the High Capacity cDNA transcription kit (ThermoFisher Scientific) at 37°C for 2 h, using random primers, and total RNA as template. For qPCRs TaqMan gene expression assays (Applied Biosystems) specific for human IFI6 (Hs00242571_m1), human IFIT2 (Hs00533665_m1), human IFNL1 (Hs00601677_g1), and human GAPDH (Hs02786624_g1) genes, were used. Data from qPCR was analysed following threshold cycle (2^-ΔΔ^ *^CT^*) methodology (34) and normalized with GAPDH expression levels, using the TaqMan gene expression assay Hs02786624_g1.

### RNAseq

HAP-1 cells specifically knocked-out for IFI6 and their parental cells were seeded. Then, cells were transfected with 1,500 ng/ml of polyinosinic-polycytidylic acid (poly(I:C), Sigma), using polyethylenimine (PEI, Polysciences) for 16 h. Total RNAs, in duplicates, were extracted using the total RNA kit (Omega Biotech), analysed in a Agilent Bioanalyzer 2100 system and sent to Novogene Co, Ltd for further RNAseq analysis. mRNA was purified from total RNA using poly-T oligo-attached magnetic beads. Fragmentation was carried out using divalent cations under elevated temperature in NEBNext First Strand Synthesis Reaction Buffer (5X). First strand cDNA was synthesized using random hexamer primer and M-MuLV Reverse Transcriptase (RNase H-). Second strand cDNA synthesis was subsequently performed using DNA Polymerase I and RNase H. Remaining overhangs were converted into blunt ends *via* exonuclease/polymerase activities. After adenylation of 3’ ends of DNA fragments, NEBNext Adaptor with hairpin loop structure were ligated to prepare for hybridization. In order to select cDNA fragments of preferentially 150~200 bp in length, the library fragments were purified with AMPure XP system (Beckman Coulter, Beverly, USA). Then 3 μl USER Enzyme (NEB, USA) was used with size-selected, adaptor-ligated cDNA at 37°C for 15 min followed by 5 min at 95°C before PCR. Then PCR was performed with Phusion High-Fidelity DNA polymerase, Universal PCR primers and Index (X) Primer. At last, PCR products were purified (AMPure XP system) and library quality was assessed on the Agilent Bioanalyzer 2100 system. The clustering of the index-coded samples was performed on a cBot Cluster Generation System using TruSeq PE Cluster Kit v3-cBot-HS (Illumina) according to the manufacturer’s instructions. After cluster generation, the library preparations were sequenced on an Illumina Hiseq platform and 125 bp/150 bp paired-end reads were generated. RNA-seq data have been deposited in the NCBI’s Sequence Read Archive (SRA) database under BioProject PRJNA916250.

Differential expression analysis of two biological replicates per condition was performed using the DESeq R package (1.18.0). DESeq provide statistical routines for determining differential expression in digital gene expression data using a model based on the negative binomial distribution. The resulting p-values were adjusted using the Benjamini and Hochberg’s approach for controlling the false discovery rate. Genes with an adjusted p-value <0.05 found by DESeq were assigned as differentially expressed. Gene Ontology (GO) enrichment analysis of differentially expressed genes was implemented using the webpage (http://geneontology.org/). GO terms with corrected False Discovery rates (FDR) adjusted p-values less than 0.05 were considered significantly enriched by differential expressed genes.

### Mice experiments

Female 6-week-old C57BL/6 mice were purchased from Envigo and maintained in the animal care facility at the National Center for Biotechnology in a pathogen-free environment. The protocols involving mice were approved by the CSIC ethics committee for animal experimentation and by the Division of Animal Protection of the regional government of Madrid, according to the National and European Union legislation (PROEX89.5/20). Mice were slightly anesthetized with isoflurane and then, intranasally inoculated with 2,000 FFU/mice of the recombinant viruses. Virus replication was evaluated by assessing viral titers in the lungs at 1 and 2 dpi (n=4 per group). To that end, mice were sacrificed and the right lung lobules were extracted and homogenized. Virus titers were determined by immunofocus assay on MDCK cells as specified above. Levels of IFIT2, IFNL3, TNF and CCL2 induction were analysed in lungs at 1 and 2 dpi. To that end, the left lung lobules were extracted and incubated in RNAlater (Ambion) at 4°C during 24 h prior to adding the lungs to RNA lysis buffer, and homogenizing the lungs manually using a dounce homogenizer. Total RNA was extracted from homogenized lungs using the total RNA kit (Omega Biotech). RT reactions were performed during 2 h at 37°C, using the high capacity cDNA transcription kit and random hexamers (ThermoFisher Scientific) to generate the cDNAs. For qPCRs Taqman gene expression assays (Applied Biosystems) specific for the murine IFIT2 (Mm00492606_m1), IFNL3 (Mm00663660_g1), CCL2 (Mm00441242_m1) and TNF (Mm00443258_m1) genes, and specific for human GAPDH (Mm99999915_g1) gene, were used. Data from qPCR was analyzed following threshold cycle (2^-ΔΔ^ *^CT^*) methodology (34) and normalized with GAPDH expression levels.

### Western blots

Cells were lysed in passive lysis buffer (Promega) and clarified by centrifugation. Cell lysates or recombinant IFI6 protein were mixed with Laemmli sample buffer containing 2.5% β-mercaptoethanol, and heated at 95°C for 5 min, before SDS-PAGE electrophoresis. Proteins were transferred to nitrocellulose membranes (Biorad), and detected using primary rabbit polyclonal antibodies (pAbs) specific for the HA epitope tag (Sigma Aldrich H6908), GST epitope tag (Sigma Aldrich A7340), IFI6 (ABclonal A6157), IFI6 (St John´s laboratory STJ27910 and STJ 191871) and mouse monoclonal antibodies (mAbs) against the FLAG epitope tag (Sigma-Aldrich F3165), His epitope tag (ThermoFisher Scientific MA1-21315), GFP (Merck 11814460001), actin (Sigma-Aldrich A1978), and ubiquitin (Santa Cruz Biotechnology, sc-166553); following by binding to goat anti-rabbit (pAb) or anti-mouse (mAb) IgG antibodies (Abs) conjugated to horseradish peroxidase (Sigma-Aldrich), at a 1:4,000 dilution. Nitrocellulose membranes were revealed by chemiluminescence with the SuperSignal west femto maximum sensitivity substrate (ThermoFisher Scientific), according to the manufacturer’s recommendations. Where indicated, protein bands have been quantified by densitometry using the ImageJ (Fiji) software.

### Binding of IFI6 to poly(I:C)

Human 293T cells (6-well plate format) were transiently transfected with plasmids expressing IFI6, PRKRA, and GFP (pCAGGS-IFI6-HA, pCAGGS-FLAG-PRKRA, and pCAGGS-GFP, respectively), using lipofectamine 3000 for 24 h. Cells were lysed in Co-IP buffer (NaCl 250 mM; EDTA 1 mM; 50 mM TrisHCl, pH 7.5; NP-40 0.5%) containing protease (ThermoFisher Scientific) and phosphatase (Merck) inhibitors. In addition, a purified, recombinant IFI6 protein with N-terminal GST and C-terminal 6xHis tag, expressed in *E. coli* (Origene) was used. To prepare poly(I:C)-conjugated agarose beads, 6 mg of poly(C)-conjugated agarose beads (Sigma) per sample were washed with Tris-Buffered Saline (TBS) buffer (25 mM Tris, 150 mM NaCl) during five times. The beads were then resuspended in buffer containing 50 mM Tris and 50 mM NaCl and incubated overnight with 120 μg of inosinic acid (Sigma). Afterwards, the beads were washed twice with TBS, resuspended in TBS buffer containing 1 mM EDTA and 0.5% Triton X-100, and incubated at 4°C for 2 h with the cellular extracts expressing IFI6, PRKRA, or GFP; or with the recombinant IFI6 protein (Origene). The mixture was washed 4 times with TBS buffer containing 1 mM EDTA and 0.1% Tween 20, and the bound proteins were eluted in loading buffer at 95°C during 5 min. The eluted proteins were analyzed by Western blotting using Abs, as described before.

### Immunoprecipitation assays

Human 293T cells (100 mm plates) were transiently transfected with plasmids expressing IFI6 fused to an HA epitope tag (pCAGGS-IFI6-HA), and RIG-I fused to a FLAG epitope tag (pCAGGS-RIG-I-FLAG) using lipofectamine 3000 for 24 h. The total amount of transfected DNA plasmid was maintained constant with empty pCAGGS plasmid. Then, the cells were transfected with poly(I:C) (3,000 ng/ml) using PEI for an additional 24 h or the cells were infected with SeV during 24 h, and the cells were lysed in Co-IP buffer (NaCl 250 mM; EDTA 1 mM; 50 mM TrisHCl, pH 7.5; NP-40 0.5%) containing protease and phosphatase inhibitors. Where indicated, cellular lysates were treated with RNaseA (30 U/ml), RNase T1 (1,200 U/ml) and/or RNAse III (30 U/ml), during 60 min at 37°C, as previously reported (35). Cleared cell lysates were incubated overnight at 4°C with 30 μl of anti-FLAG affinity resin (Sigma-Aldrich, A2220). After washing three times in TBS buffer containing 0.1% tween-20 (for anti-HA resin) or TBS containing 0.1% SDS (for anti-FLAG resin), precipitated proteins were dissociated using 0.1 M glycine buffer at pH 2.4, denaturalized in loading buffer and incubated at 95°C during 5 min. Then, samples were analyzed by Western blot as described above using anti-HA (IFI6), and anti-FLAG (RIG-I) specific Abs.

### Immunofluorescence and confocal microscopy

Confluent monolayers of human A549 cells on sterile glass coverslips (24-well plate format) were transiently transfected with pCAGGS plasmids expressing RIG-I-FLAG and IFI6-HA using lipofectamine 3000. At 24 hpt, cells were transfected with poly(I:C). Alternatively, confluent monolayers of MDCK cells were mock-infected or infected (MOI 0.01) with mCherry- or IFI6-expressing IAV. At 24 h after poly(I:C) transfection or at 24 hours post-infection (hpi), the cells were fixed and permeabilized with 10% formaldehyde and 0.1% Triton X100 for 20 min at room temperature. Then, cells were blocked with 2.5% BSA in PBS and RIG-I-FLAG, IFI6-HA and IAV NP were detected with murine anti-FLAG and rabbit anti-HA pAbs, and with a mouse Ab for IAV NP (mAb HB-65, ATCC H16-L10-4R), respectively. Coverslips were washed with PBS for 4 times, and incubated with secondary anti-mouse and anti-rabbit Abs conjugated to Alexa Fluor 488 and 546 (Invitrogen), during 45 min at room temperature. Nuclei were stained using DAPI (ThermoFisher Scientific). Coverslips were mounted in ProLong Gold antifade reagent (Invitrogen) and analyzed on a Leica STELLARIS 5 confocal microscope. Images were acquired with the same instrument settings and analyzed using the Fiji software.

### RIG-I ubiquitination and overexpression assays

Human 293T cells (6-well plate format) were transfected with siRNAs specific for human IFI6 (ThermoFisher Scientific, s5441), or with the NT negative control siRNA. At 24 hpt, cells were transfected with the plasmid pCAGGS-RIG-I-FLAG and 24 h later the cells were infected with SeV (MOI 3) for an additional 24 h. Then, cells were lysed in the Co-IP buffer, containing protease and phosphatase inhibitors and the cellular lysates were subjected to anti-FLAG immunoprecipitation as described above in the “immunoprecipitation assays”. Immunoprecipitated proteins were assayed with the anti-FLAG and anti-ubiquitin Abs. To assess induction of innate immune responses mediated by RIG-I, human 293T cells (24-well plate format) were transfected with pCAGGS-RIG-I-FLAG and pCAGGS-IFI6-HA plasmids and 24 h later cells were infected with SeV (MOI 3) for an additional 24 h. Total RNAs were extracted using the total RNA extraction kit (Omega Biotek), and expression of IFNL1 was analyzed by RT-qPCR, as specified above.

## Results

### IFN and viral infections induce the expression of IFI6

**Figure 1:**
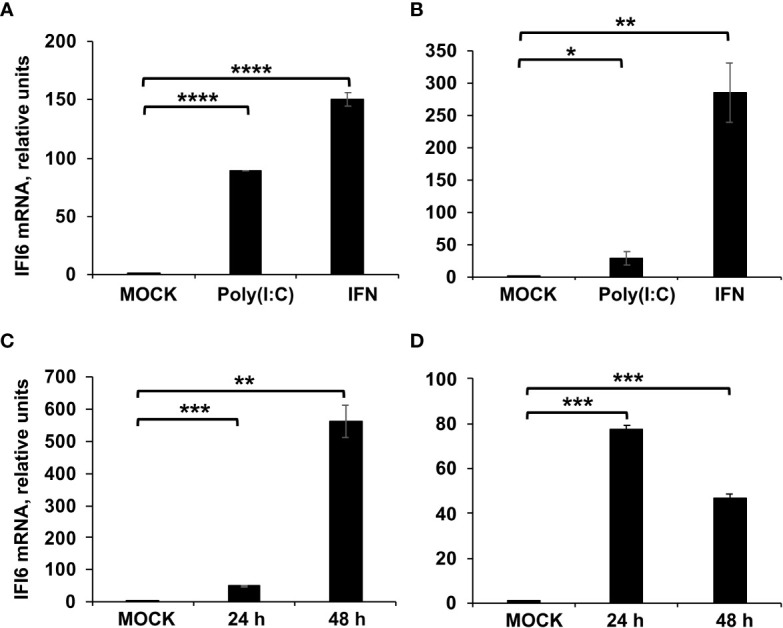
IFI6 expression is induced by poly(I:C) transfection, IFN treatment, and IAV and SARS-CoV-2 infection. **(A, B)** A549 and HAP-1 cells, respectively, were transfected with poly(I:C) or treated with IFN during 16 h. **(C)** A549 cells were infected with IAV (MOI 3) during 24 and 48 hpi. **(D)** A549 cells overexpressing hACE-2 were infected with SARS-CoV-2 (MOI 1) during 24 and 48 hpi. **(A–D)** The levels of IFI6 mRNAs were evaluated by RT-qPCR and compared to the levels in non-treated or non-infected cells (mock). Error bars represent standard deviations (SD) of results of measurements performed in triplicate wells. *P< 0.05, **P<0.01, ***P<0.001, ****P<0.0001 using an Student’s t test.

IFI6 (also known as G1P3, ISG16, or IFI6-16) has been previously described as an IFN-induced gene (36, 37), which is induced by infection of many RNA viruses such as influenza (38), flaviviruses (15, 17) and SARS-CoV-2 (39). To confirm that IFI6 is an ISG in our cell culture systems, epithelial human lung adenocarcinoma-derived A549 cells were treated with poly(I:C), an analog of dsRNA, or with IFNα, for 16 h, and the levels of IFI6 were measured by RT-qPCR, and compared to the levels in mock-treated cells. The IFI6 mRNA levels were increased by ~90 and ~150-fold in poly(I:C) and IFNα-treated cells, respectively (**Figure 1A**). To confirm that IFI6 was also induced in cells from other origins, the human leukemia-derived HAP-1 cells were treated with poly(I:C) (2,000 ng/ml) and IFNα (2,000 units/ml) for 16 h. The levels of IFI6 mRNA were measured showing increases of ~28 and ~284-fold, respectively, in comparison to mock-infected cells (**Figure 1B**), confirming that IFI6 behaves as an ISG in our cell culture systems.

To analyze whether IFI6 is induced after the infection with respiratory viruses, such as influenza and coronaviruses, lung adenocarcinoma-derived A549 and A549 cells overexpressing the SARS-CoV-2 receptor hACE-2, were infected with IAV (MOI 3) or SARS-CoV-2 (MOI 1), respectively. The levels of IFI6 mRNA were evaluated, showing increases of ~50 and ~80-fold at 24 hpi, and increases of ~550 and ~50-fold at 48 hpi after IAV and SARS-CoV-2 infection, respectively (**Figures 1C, D**), indicating that IFI6 expression is induced after IAV and SARS-CoV-2 infections. We tried to show that IFI6 is upregulated at the protein level by Western blot. Unfortunately, we tried several commercial antibodies against IFI6, and we could not detect the IFI6 protein, even in cell extracts overexpressing the IFI6 protein (data not shown).

### Blocking expression of IFI6 negatively affects IAV and SARS-CoV-2 production

**Figure 2:**
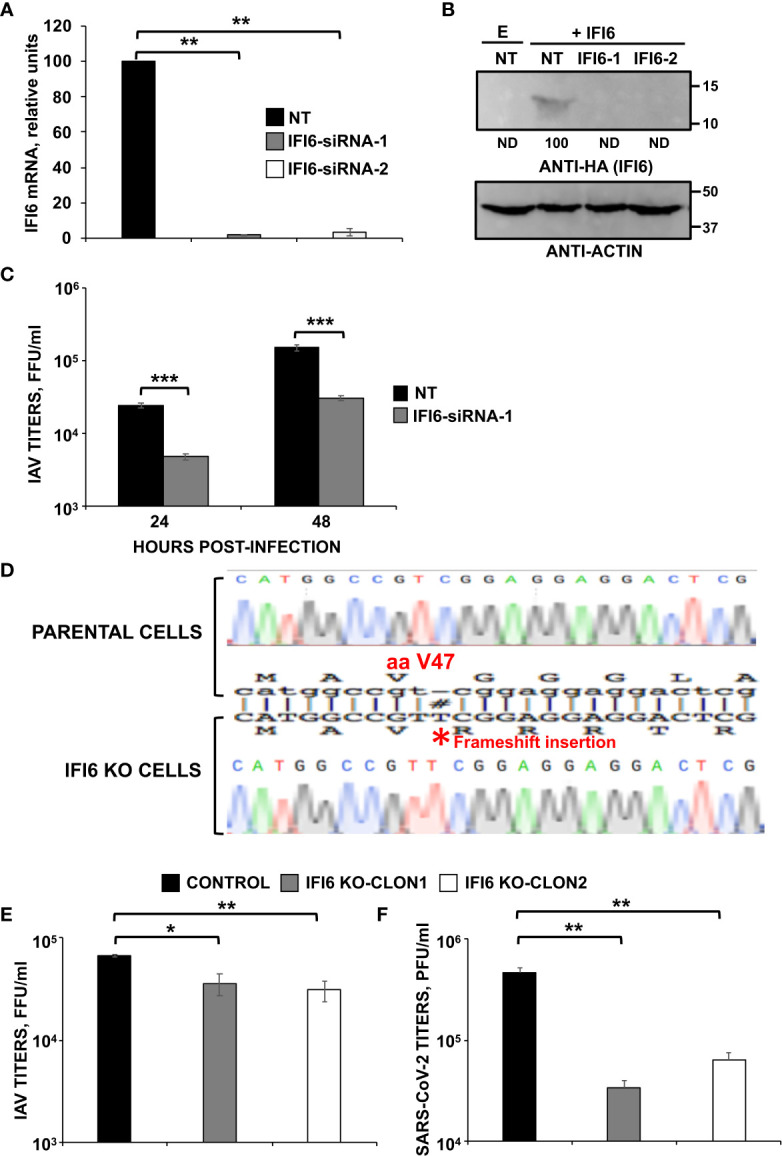
IFI6 silencing and knocking-out negatively affects viral infections. *Silencing results.* **(A–C)** Human A549 cells were transfected with a control, non-targeted (NT) or IFI6 siRNAs. **(A)** At 24 hpt, total RNAs were purified and used to determine the mRNA levels for IFI6 by RT-qPCR. **(B)** At 24 h after siRNA transfection, cells were transfected with the plasmid expressing IFI6 fused to an HA tag of with an empty plasmid, as control, during 24 h. A Western blot analysis using anti-HA antibodies (to detect IFI6; top) and anti-actin antibodies (bottom) was performed. Molecular weight markers are indicated (in kDa) on the right. Western blots after the IP were quantified by densitometry using ImageJ software, and the amounts of IFI6-HA were normalized to the amounts of actin (numbers below the top blot). ND, not detected. Three different experiments were performed, with similar results. **(C)** At 24 hpt, cells were infected with IAV. At 24 and 48 hpi, cell culture supernatants were collected and titrated by immunofocus assay. Three different experiments were performed, with similar results. **P<0.01, ***P<0.001 (for comparisons between NT- and IFI6-silenced cells at 24 and 48 hpi using Student´s *t* test). *Knocking-out results*. **(D)** Generation of IFI6 knock-out (KO) cells. Sequencing of A549 parental cells and IFI6 A549 KO cells, showing the insertion of 1 nucleotide within the open reading frame of IFI6 (bottom), in comparison to parental cells (top). The amino acid position (40) at which the insertion occurred is indicated, #, indicates an insertion within the sequence alignment. **(E)** Distinct clones of A549 cells specifically KO for the IFI6 gene were infected with IAV. Viral titers in cell culture supernatants were measured at 24 hours post-infection (hpi) by immunofocus assay in MDCK cells (fluorescence forming units per ml, FFU/ml) and compared to the titers in the parental, control cells. **(F)** A549 clones specifically KO for IFI6 and overexpressing hACE-2, were infected with SARS-CoV-2. Viral titers in cell culture supernatants were measured at 48 hpi, by a plaque assay in Vero E6 cells (plaque forming units per ml, PFU/ml), and compared to the titers in the parental, control cells. Three different experiments were performed, with similar results. *P< 0.05, **P<0.01, (for comparisons between parental IFI6 KO cells using Student´s *t* test).

To study whether IFI6 modulates IAV and SARS-CoV-2 replication, first, we silenced the expression of IFI6 in the epithelial A549 cells by transfecting the cells with two different siRNAs or with a non-targeted (NT) siRNA, as control. The expression of IFI6 was silenced by more than 90% at the mRNA level using both siRNAs, as measured by RT-qPCR (**Figure 2A**). To confirm that expression of IFI6 was silenced at the protein level as well, A549 cells were transfected with siRNAs, and at 24 hpt, cells were transfected with a plasmid expressing IFI6 containing a C-terminal HA epitope tag, or empty plasmid, for an additional 24 h. Western blots using an anti-HA epitope specific Ab, and an anti-actin Ab as loading control, showed that whereas IFI6 protein could be detected in cells transfected with the non-targeted (NT) control siRNA, no IFI6 protein could be detected in cells transfected with the two IFI6 specific siRNAs (**Figure 2B**), confirming that transfection of cells with the IFI6 specific siRNAs efficiently knocks down the expression of IFI6.

To assess whether IFI6 silencing had an effect on IAV production, A549 cells were silenced with one of the IFI6-specific siRNA, or the NT control siRNA, and then infected (MOI 3) with IAV. A reproducible and significant ~8 and ~7-fold-reduction IAV viral titers was observed in cells silenced with the IFI6 specific siRNA compared to NT siRNA-silenced A549 cells at 24 and 48 hpi, respectively (**Figure 2C**). These data suggest that IFI6 silencing negatively affects IAV production.

To confirm these initial results in siRNA silenced cells, epithelial A549 knock-out (KO) cells for IFI6 were generated using CRISPR/Cas9 technologies. To this end, we used a pX330 plasmid encoding a sgRNA, the gene for caspase 9, and a puromycin-resistance gene, to select the transfected cells in the presence of the antibiotic. Different clones were obtained by cell limiting dilution, and two clones were selected for our studies. The different clones obtained encoded a 1-nucleotide insertion, leading to a frameshift starting from amino acid 47 of the protein (**Figure 2D**).

To further confirm that IFI6 negatively modulates IAV replication, the parental cells and two independently isolated clones of IFI6 KO A549 cells were infected with IAV (MOI 3) and viral titers were measured at 24 hpi. An ~2 to 3-fold decrease in viral titers was observed in IFI6 KO cells compared to the parental cells (**Figure 2E**), consistent with our results using IFI6 siRNAs (**Figure 2C**).

To ascertain whether the effect of IFI6 also applies to other viruses, we transduced our A549 parental cells, and two clones of A549 IFI6 KO cells with a retrovirus which expresses hACE-2, so that the cells become permissive to SARS-CoV-2 infection. These cells were infected with SARS-CoV-2 (MOI 1) and viral titers were decreased by ~6 to 10-fold in IFI6 KO cells compared to the parental cells (**Figure 2F**). These results indicated that the knocking-down or suppression of IFI6 expression decreased IAV and SARS-CoV-2 titers, two different unrelated human respiratory viruses.

### Effect of IFI6 on antiviral responses

Many ISGs downregulate antiviral responses through a negative feedback mechanism (7, 10, 11, 41), as exacerbated innate immune responses can be deleterious for the host. Taking this information into account, and the fact that IAV and SARS-CoV-2 are unrelated viruses which are sensitive to IFN responses (42, 43), we hypothesized that IFI6 could be modulating the induction of the host antiviral responses. To test this hypothesis and to get a broader insight on the effect of IFI6 on host responses, the transcriptomes of human leukemia-derived HAP-1 control cells and IFI6 KO cells were compared by RNAseq. First, the genes differentially upregulated at least 2-fold, with a false discovery rate (FDR) adjusted p-value<0.05 in HAP-1 control cells transfected with poly(I:C), an analog of dsRNA which is produced during the infection with many viruses, compared to non-transfected control cells, were classified by gene ontology according to the “biological process” they are involved in (**Tables 1**, **2**). These genes are classified in terms such as: negative regulation of viral genome replication, positive regulation of IFN-beta production, interleukin 27-mediated signaling pathway, antiviral innate immune response, regulation of defense response to virus, cytoplasmic pattern recognition receptor signaling pathway in response to virus, positive regulation of RIG-I signaling pathway, cellular response to interferon-alpha, cellular response to exogenous dsRNA, response to bacterium, positive regulation of interferon-alpha production, positive regulation of tumor necrosis factor production, positive regulation of MDA-5 signaling pathway, type I interferon signaling pathway, regulation of type III interferon production, ISG15-protein conjugation, regulation by virus of viral protein levels in host cell, negative regulation of cytokine-mediated signaling pathway, and negative regulation of innate immune response (**Table 1**), indicating that poly(I:C) transfection induces innate immune responses.

**Table 1:** Genes differentially upregulated (at least 2-fold) in HAP-1, control, poly(I:C)-treated cells compared to HAP-1, control, mock-treated cells, classified using Gene Ontology according to the biological process they are involved in.

GENE ONTOLOGY TERMS	Homo sapiens REFLIST: 20589 genes	Genes upregulated in the RNAseq experiment: 28	Number of expected genes	Fold Enrichment in the uploaded upregulated genes	FDR adjusted p- value
**negative regulation of viral genome replication (GO:0045071)**	57	8	0.07	> 100	1.48E-11
**positive regulation of interferon-beta production (GO:0032728)**	41	6	0.05	> 100	2.05E-08
**interleukin-27-mediated signaling pathway (GO:0070106)**	7	4	0.01	> 100	4.44E-07
**antiviral innate immune response (GO:0140374)**	22	4	0.03	> 100	1.62E-05
**regulation of defense response to virus (GO:0050688)**	76	5	0.1	50.17	2.79E-05
regulation of ribonuclease activity (GO:0060700)	9	3	0.01	> 100	1.76E-04
**cytoplasmic pattern recognition receptor signaling pathway in response to virus (GO:0039528)**	11	3	0.01	> 100	2.70E-04
**positive regulation of RIG-I signaling pathway (GO:1900246)**	11	3	0.01	> 100	2.76E-04
**cellular response to interferon-alpha (GO:0035457)**	12	3	0.02	> 100	3.29E-04
**cellular response to exogenous dsRNA (GO:0071360)**	19	3	0.02	> 100	9.71E-04
**response to bacterium (GO:0009617)**	754	8	0.99	8.09	1.18E-03
**positive regulation of interferon-alpha production (GO:0032727)**	25	3	0.03	91.51	1.74E-03
**positive regulation of tumor necrosis factor production (GO:0032760)**	101	4	0.13	30.2	2.50E-03
**positive regulation of MDA-5 signaling pathway (GO:1900245)**	4	2	0.01	> 100	5.53E-03
**type I interferon signaling pathway (GO:0060337)**	42	3	0.06	54.47	6.05E-03
**regulation of type III interferon production (GO:0034344)**	5	2	0.01	> 100	7.52E-03
**ISG15-protein conjugation (GO:0032020)**	6	2	0.01	> 100	9.75E-03
**regulation by virus of viral protein levels in host cell (GO:0046719)**	8	2	0.01	> 100	1.43E-02
**negative regulation of cytokine-mediated signaling pathway (GO:0001960)**	75	3	0.1	30.5	2.66E-02
**negative regulation of innate immune response (GO:0045824)**	76	3	0.1	30.1	2.73E-02

Gene ontology terms related to innate immune responses are indicated in bold.

**Table 2:** Genes differentially upregulated (at least 2-fold) in HAP-1, IFI6 KO, poly(I:C)-treated cells compared to HAP-1, control, poly(I:C)-treated cells, classified using Gene Ontology according to the biological process they are involved in.

Gene Ontology terms	Homo sapiens REFLIST: 20589 genes	Genes upregulated in the RNAseq experiment: 1204	Number of expected genes taking into account the number of upregulated genes in the RNAseq experiment	Fold Enrichment in the uploaded upregulated genes	FDR value
**negative regulation of viral process (GO:0048525)**	95	31	5.56	5.58	6.38E-10
**negative regulation of viral genome replication (GO:0045071)**	57	23	3.33	6.9	1.18E-08
**defense response to virus (GO:0051607)**	253	47	14.79	3.18	3.08E-08
**response to virus (GO:0009615)**	357	54	20.88	2.59	9.63E-07
**regulation of viral genome replication (GO:0045069)**	87	23	5.09	4.52	5.07E-06
angiogenesis (GO:0001525)	327	49	19.12	2.56	5.78E-06
**positive regulation of cell migration (GO:0030335)**	532	64	31.11	2.06	4.98E-05
regulation of epithelial cell proliferation (GO:0050678)	369	47	21.58	2.18	3.79E-04
**negative regulation of cell migration (GO:0030336)**	288	39	16.84	2.32	6.79E-04
cellular response to fibroblast growth factor stimulus (GO:0044344)	85	18	4.97	3.62	1.18E-03
**regulation of response to external stimulus (GO:0032101)**	973	93	56.9	1.63	1.20E-03
**regulation of MAP kinase activity (GO:0043405)**	184	28	10.76	2.6	1.92E-03
regulation of animal organ morphogenesis (GO:2000027)	129	22	7.54	2.92	2.39E-03
**response to interferon-alpha (GO:0035455)**	23	9	1.34	6.69	3.08E-03
collagen fibril organization (GO:0030199)	60	14	3.51	3.99	3.44E-03
**response to type I interferon (GO:0034340)**	52	13	3.04	4.28	3.46E-03
regulation of peptidyl-tyrosine phosphorylation (GO:0050730)	261	34	15.26	2.23	3.62E-03
negative regulation of fibroblast growth factor receptor signaling pathway (GO:0040037)	18	8	1.05	7.6	3.88E-03
regulation of vesicle-mediated transport (GO:0060627)	552	58	32.28	1.8	4.44E-03
response to hormone (GO:0009725)	767	74	44.85	1.65	5.16E-03
response to hypoxia (GO:0001666)	277	35	16.2	2.16	5.20E-03
regulation of axon extension involved in axon guidance (GO:0048841)	33	10	1.93	5.18	5.90E-03
cell-cell adhesion via plasma-membrane adhesion molecules (GO:0098742)	267	34	15.61	2.18	6.28E-03
peptidyl-lysine oxidation (GO:0018057)	5	5	0.29	17.1	6.39E-03
canonical Wnt signaling pathway (GO:0060070)	102	18	5.96	3.02	6.47E-03
**positive regulation of interferon-beta production (GO:0032728)**	41	11	2.4	4.59	6.48E-03
regulation of Wnt signaling pathway (GO:0030111)	332	39	19.41	2.01	7.43E-03
regulation of transmembrane receptor protein serine/threonine kinase signaling pathway (GO:0090092)	275	34	16.08	2.11	7.77E-03
gastrulation with mouth forming second (GO:0001702)	28	9	1.64	5.5	8.30E-03
negative regulation of cell-substrate adhesion (GO:0010812)	60	13	3.51	3.71	9.37E-03
regulation of blood coagulation (GO:0030193)	70	14	4.09	3.42	1.09E-02
positive regulation of cell death (GO:0010942)	583	58	34.09	1.7	1.16E-02
**response to interferon-gamma (GO:0034341)**	130	20	7.6	2.63	1.25E-02
BMP signaling pathway (GO:0030509)	91	16	5.32	3.01	1.39E-02
positive regulation of ossification (GO:0045778)	55	12	3.22	3.73	1.46E-02
**interleukin-27-mediated signaling pathway (GO:0070106)**	7	5	0.41	12.21	1.51E-02
**negative chemotaxis (GO:0050919)**	47	11	2.75	4	1.51E-02
positive regulation of axon extension (GO:0045773)	39	10	2.28	4.38	1.52E-02
regulation of angiogenesis (GO:0045765)	288	34	16.84	2.02	1.55E-02
glomerulus development (GO:0032835)	56	12	3.27	3.66	1.60E-02
response to retinoic acid (GO:0032526)	113	18	6.61	2.72	1.62E-02
regulation of synapse assembly (GO:0051963)	103	17	6.02	2.82	1.63E-02
**regulation of viral entry into host cell (GO:0046596)**	48	11	2.81	3.92	1.69E-02
positive regulation of neural precursor cell proliferation (GO:2000179)	57	12	3.33	3.6	1.79E-02
regulation of phosphatidylinositol 3-kinase activity (GO:0043551)	57	12	3.33	3.6	1.79E-02
metanephros development (GO:0001656)	85	15	4.97	3.02	1.87E-02
regulation of Notch signaling pathway (GO:0008593)	95	16	5.56	2.88	1.91E-02
**response to interferon-beta (GO:0035456)**	33	9	1.93	4.66	1.92E-02
regulation of apoptotic process (GO:0042981)	1468	120	85.85	1.4	1.92E-02
cell-substrate junction organization (GO:0150115)	41	10	2.4	4.17	1.97E-02
**regulation of ERK1 and ERK2 cascade (GO:0070372)**	303	35	17.72	1.98	1.97E-02
**negative regulation of MAP kinase activity (GO:0043407)**	58	12	3.39	3.54	1.98E-02
regulation of epithelial to mesenchymal transition (GO:0010717)	97	16	5.67	2.82	2.22E-02
negative regulation of vascular permeability (GO:0043116)	20	7	1.17	5.99	2.39E-02
negative regulation of axon extension involved in axon guidance (GO:0048843)	27	8	1.58	5.07	2.42E-02
negative regulation of wound healing (GO:0061045)	69	13	4.03	3.22	2.45E-02
negative regulation of endothelial cell proliferation (GO:0001937)	43	10	2.51	3.98	2.58E-02
**negative regulation of viral life cycle (GO:1903901)**	28	8	1.64	4.89	2.87E-02
**cellular response to type I interferon (GO:0071357)**	44	10	2.57	3.89	2.95E-02
regulation of ribonuclease activity (GO:0060700)	9	5	0.53	9.5	2.99E-02
**regulation of immune system process (GO:0002682)**	1520	122	88.89	1.37	3.19E-02
endocardial cushion morphogenesis (GO:0003203)	37	9	2.16	4.16	3.43E-02
regulation of response to biotic stimulus (GO:0002831)	364	39	21.29	1.83	3.56E-02
**regulation of tumor necrosis factor production (GO:0032680)**	163	22	9.53	2.31	3.72E-02
aorta morphogenesis (GO:0035909)	30	8	1.75	4.56	3.90E-02
neutrophil homeostasis (GO:0001780)	16	6	0.94	6.41	4.00E-02
heart valve development (GO:0003170)	65	12	3.8	3.16	4.11E-02
hair follicle morphogenesis (GO:0031069)	31	8	1.81	4.41	4.49E-02
**negative regulation of viral entry into host cell (GO:0046597)**	24	7	1.4	4.99	4.94E-02

Gene ontology terms related to innate immune responses are indicated in bold.

**Figure 3:**
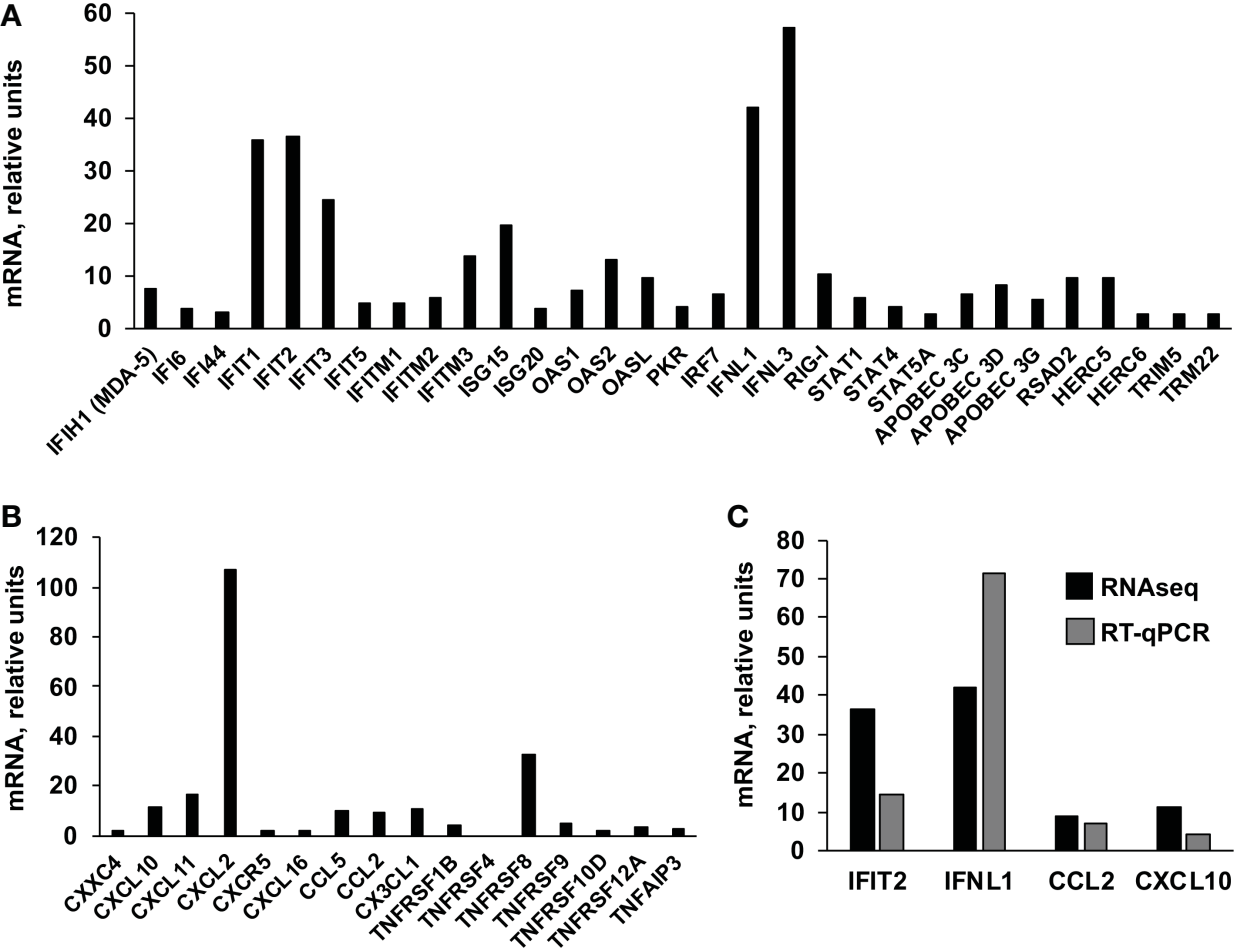
Effect of IFI6 on host gene expression. HAP-1 control and IFI6 KO cells were either mock-treated or treated with poly(I:C). Total RNAs were extracted and the cellular transcriptomes were analyzed by RNAseq. The ISGs **(A)** and genes encoding proinflammatory cytokines **(B)** which are differentially upregulated in HAP-1, IFI6 KO, poly(I:C)-treated cells compared to HAP-1, control, poly(I:C)-treated cells, are represented. **(C)** Comparison of the levels of expression of IFT2 (an ISG), IFNL1 (a type III IFN), CCL2 and CXCL10 (two pro-inflammatory cytokines), as measured by RNAseq and RT-qPCR.

Furthermore, we compared the transcriptome of poly(I:C)-transfected HAP-1 control cells, to the transcriptome of poly(I:C)-transfected, HAP-1 IFI6 KO cells (**Table 2**). The genes differentially upregulated at least 2-fold, with an FDR adjusted p-value <0.05 in the IFI6 KO cells, were classified according to their biological process, showing that genes involved in: negative regulation of viral process, negative regulation of viral genome replication, defense response to virus, response to virus, regulation of viral genome replication, positive regulation of cell migration, negative regulation of cell migration, regulation of response to external stimulus, regulation of MAP kinase activity, response to interferon-alpha, response to type I interferon, positive regulation of interferon-beta production, response to interferon-gamma, interleukin-27-mediated signaling pathway, negative chemotaxis, regulation of viral entry into host cell, response to interferon-beta, regulation of ERK1 and ERK2 cascade, negative regulation of MAP kinase activity, negative regulation of viral life cycle, cellular response to type I interferon, regulation of immune system process, regulation of tumor necrosis factor production, and negative regulation of viral entry into host cell cells (**Table 2**), are overrepresented in the poly(I:C)-transfected IFI6 KO cells, compared to the poly(I:C)-transfected control cells (**Table 2**), suggesting that IFI6 counteracts interferon and inflammatory cytokine responses. Furthermore, the genes related to IFN and inflammatory responses, which were differentially upregulated at least 2-fold in the poly(I:C)-transfected HAP-1 IFI6 KO cells, compared to the poly(I:C)-transfected HAP-1 control cells, were represented (**Figures 3A, B**). Type III IFN genes (IFNL1, and IFNL3), genes involved in dsRNA sensing leading to IFN signaling pathways (MDA-5, and RIG-I), transcription factors involved in IFN genes transcription (IRF-7), transcription factors involved in the transcription of ISGs (STAT1, STAT4, and STAT5A) and ISGs (IFI6, IFI44, IFIT1, IFIT2, IFIT3, IFIT5, IFITM1, IFITM2, IFITM3, ISG15, ISG20, OAS1, OAS2, OASL, PKR, APOBEC 3C, APOBEC 3D, APOBEC 3G, RSAD2, HERC5, HERC6, TRIM5, and TRIM22) were upregulated in the IFI6 KO cells transfected with poly(I:C) compared to the poly(I:C)-transfected control cells (**Figure 3A**), as measured by RNAseq. In addition, genes encoding pro-inflammatory cytokines and cytokines involved in immune cell migration (CXXC4, CXCL10, CXCL11, CXCL2, CXCL16, CCL2, CCL5, CX3CL1), genes encoding a pro-inflammatory cytokine receptor (CXCR5, receptor for CXCL13), and genes belonging to the TNF superfamily (TNFRSF1B, TNFRSF4, TNFRSF8, TNFSRF9, TNFRSF10D, TNFRSF12A, and TNFAIP3) were upregulated in the IFI6 KO cells transfected with poly(I:C) compared to the poly(I:C)-transfected control cells (**Figure 3B**), as measured by RNAseq. Furthermore, the differential of expression of some of these genes (i.e. IFIT2, IFNL1, CCL2, and CXCL10) was further confirmed by RT-qPCR, showing similar results using RNAseq and RT-qPCR, therefore, validating the RNAseq assays (**Figure 3C**), and providing the basis for analyzing the expression of these genes on subsequent experiments.

**Figure 4:**
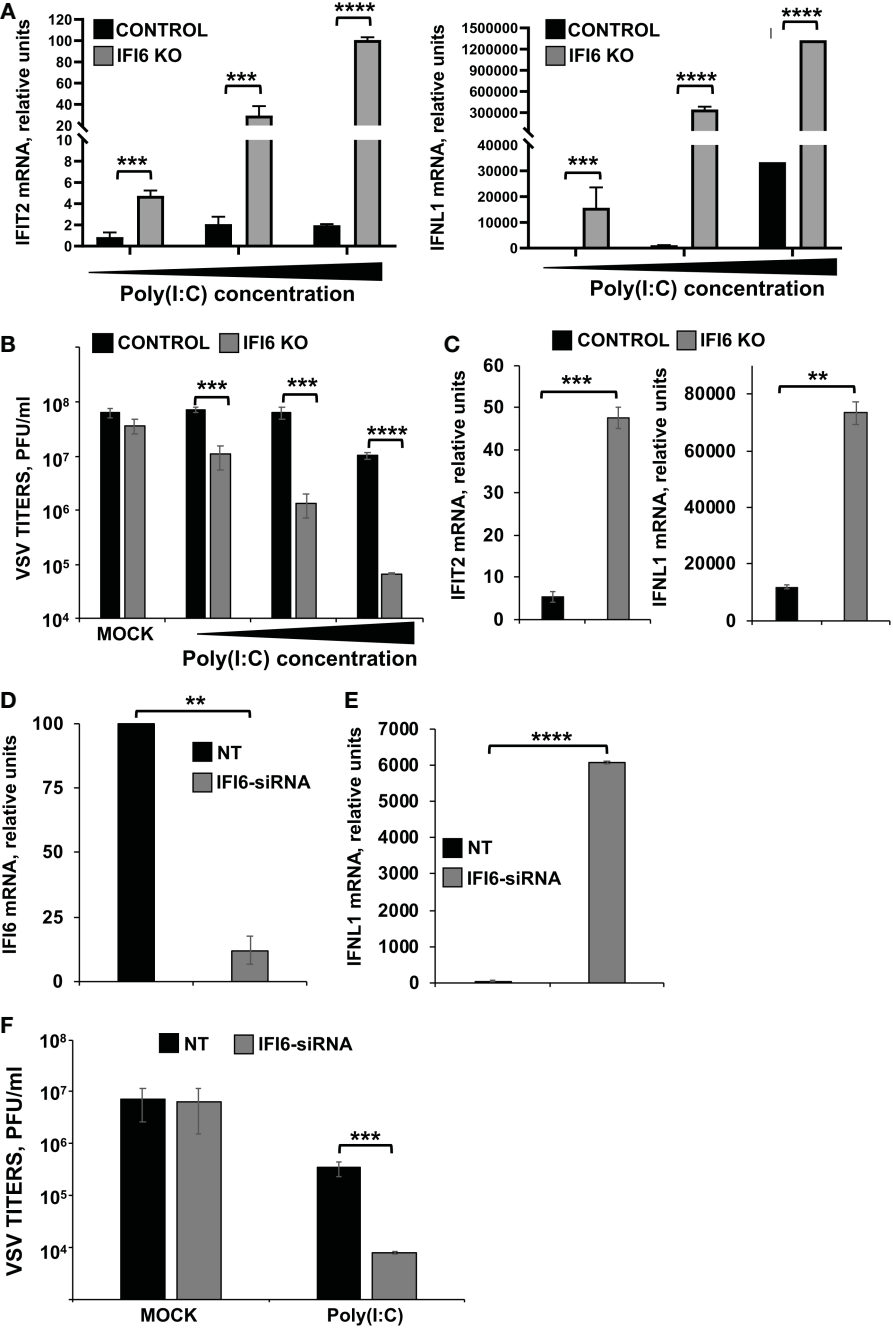
Effect of IFI6 on the modulation of innate immune responses induced by poly(I:C). **(A, B)** *Effect of IFI6 knocking-out*. **(A)** Human HAP-1 IFI6 KO and WT cells were treated with three different concentrations of poly(I:C) (750; 1,500; and 3,000 ng/ml). **(A)** To analyze the effect of IFI6 on innate immune responses, the levels of IFIT2 and IFNL1 were evaluated by RT-qPCR at 24 hpt, and mRNA levels were expressed as fold change (increases) in comparison to mock-treated cells, used as controls. **(B)** Mock-treated cells, or cells transfected with three different concentrations of poly(I:C), were infected with rVSV-GFP (MOI of 0.1), and viral titers at 24 hpi were measured by a lysis plaque assay. Three different experiments were performed, with similar results. ***P<0.001, ****P<0.0001 (using Student’s *t* test). **(C)** Human HAP-1 parental and IF6 KO cells were infected for 24 h with SeV. The levels of IFIT2, and IFNL1 were measured by RT-qPCR and mRNA levels were expressed as fold change (increases) in comparison to mock-infected cells, used as controls. **P<0.01, ***P<0.001 (using Student’s *t* test). **(D–F)** *Effect of IFI6 silencing*. **(D–F)** Human HAP-1 cells were transfected with NT or an IFI6 siRNA. **(D)** At 24 hpt, total RNAs were purified and mRNA levels for IFI6 were analyzed by RT-qPCR. **(E)** At 24 hpt, cells were transfected with poly(I:C). To analyze the effect of IFI6 on innate immune responses, the levels of IFNL1 were evaluated by RT-qPCR at 24 hpt, and increases in mRNA levels were expressed as fold change in comparison to mock-treated cells, used as controls. **(F)** Cells that had been subjected to mock treatment, or transfected with polyIC, were infected with rVSV-GFP (MOI of 0.1), and viral titers at 24 hpi were measured by a lysis plaque assay. Three different experiments were performed, with similar results. **P<0.01, ***P<0.001, ****P<0.0001 (using Student’s *t* test).

Moreover, to confirm the RNAseq results, the induction of IFIT2 and IFNL1 after poly(I:C) treatment was further compared in the parental (control) and IFI6 KO HAP-1 cells. Using three different concentrations of poly(I:C) (3,000; 1,500; and 750 ng/ml), it was shown that the treatment of these cells with poly(I:C) induced the expression of IFIT2 and IFNL1 in a dose-dependent manner (**Figure 4A**). Interestingly, these two genes were induced to higher extent in IFI6 KO HAP-1 cells than in control cells (**Figure 4A**). Moreover, poly(I:C)-transfected cells were infected with rVSV-GFP (MOI 0.1), a virus highly sensitive to the antiviral state induced by poly(I:C) (29, 44), as an indirect measure of the effect of IFI6 on the antiviral state induced in the cells. Results indicate that poly(I:C) transfection only reduced virus titers by ~6-fold using the highest poly(I:C) concentration, compared to non-transfected control HAP-1 cells. Interestingly, rVSV-GFP titers were decreased by ~1,000-fold in poly(I:C)-transfected IFI6 KO HAP-1 cells, in comparison to poly(I:C)-transfected control HAP-1 cells (**Figure 4B**). Moreover, to analyze the effect of IFI6 on HAP-1-infected cells, and since HAP-1 cells are not highly susceptible to IAV and SARS-CoV-2 infections (data not shown), HAP-1 parental and IFI6 KO cells were infected with Sendai virus (SeV). Expression of IFIT2 and IFNL1 was increased by ~9- and 7-fold, respectively, in HAP-1 IFI6 KO cells infected with SeV, as compared to parental HAP-1 SeV-infected cells (**Figure 4C**), indicating that blocking IFI6 expression increases the induction of host antiviral responses.

To confirm these results using another approach, HAP-1 cells were transfected with a siRNA specific for IFI6 to knock-down the expression of IFI6, or with a non-targeted siRNA, as control. Expression of IFI6 was downregulated by ~ 80% in IFI6 siRNA transfected HAP-1 cells compared to the NT siRNA-transfected HAP-1 cells, as determined by RT-qPCR (**Figure 4D**). In poly(I:C)-transfected cells, expression of IFNL1 was upregulated in IFI6 knocked-down cells, compared to the control cells (**Figure 4E**). In addition, and correlating with these results, rVSV-GFP titers in poly(I:C)-transfected cells were lower in IFI6 knocked-down cells than in control cells (**Figure 4F**), further confirming that IFI6 acts as a negative modulator of innate immune responses.

**Figure 5:**
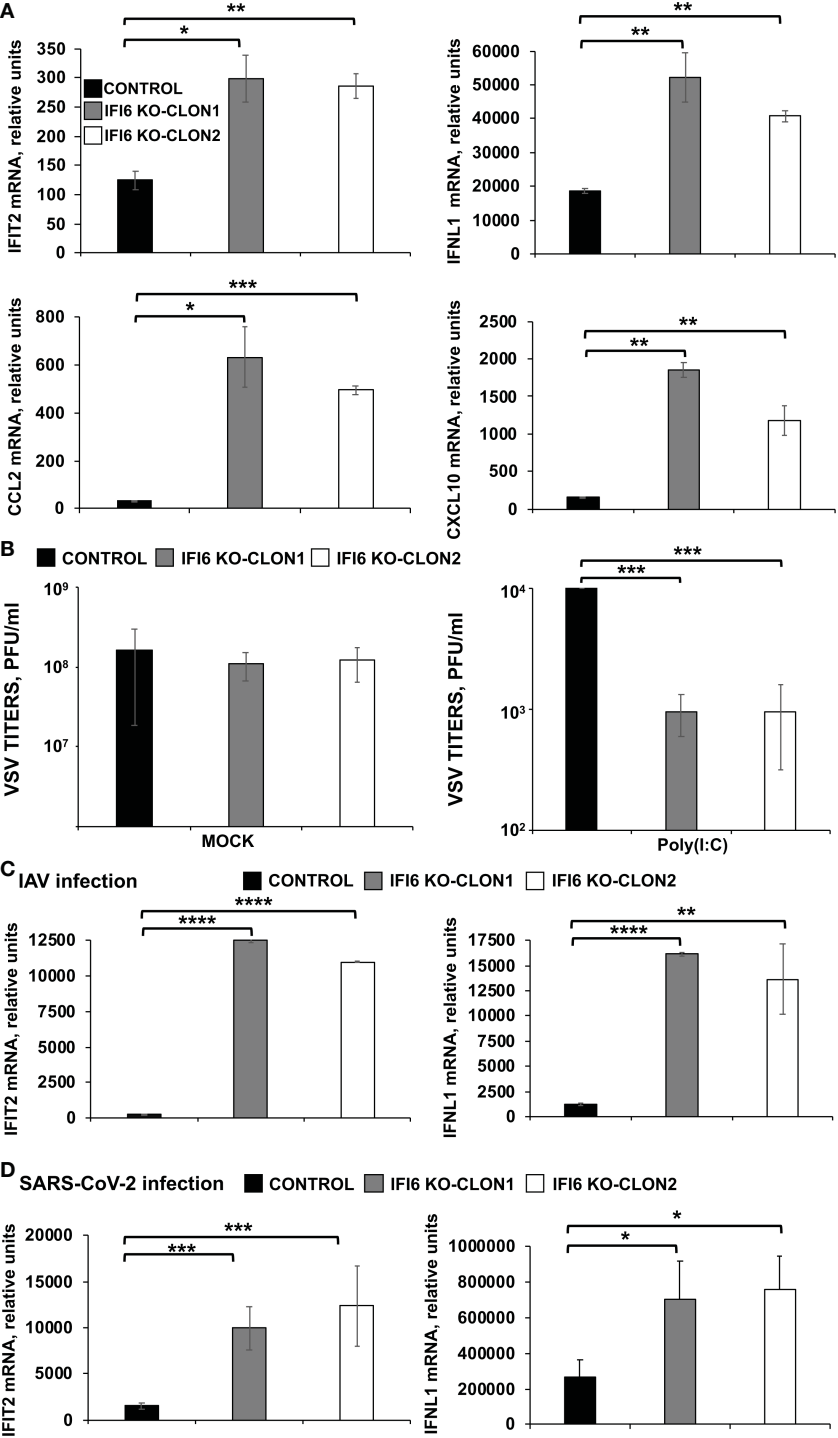
Effect of IFI6 on the modulation of innate immune responses induced by poly(I:C) and viral infections in A549 epithelial cells. **(A, B)** Two clones of A549 cells specifically KO for the gene IFI6 were treated with poly(I:C). **(A)** To analyze the effect of IFI6 on innate immune responses, the levels of IFIT2, IFNL1, CCL2, and CXCL10 were evaluated by RT-qPCR at 24 hpt, and mRNA levels were expressed as fold change (increases) in comparison to mock-treated cells, used as controls. **(B)** Cells that had been subjected to mock treatment, or transfected with poly(I:C), were infected with rVSV-GFP (MOI of 0.1), and viral titers at 24 hpi were measured by a lysis plaque assay. Three different experiments were performed, with similar results. *P< 0.05, **P<0.01, ***P<0.001 (using Student’s *t* test). **(C)** Two clones of A549 cells specifically KO for the gene IFI6 were infected with IAV during 24 h. **(D)** Two clones of A549 cells specifically KO for the gene IFI6 were infected with SARS-CoV-2 (MOI 1) during 48 h [59–62]. **(C, D)** The levels of IFIT2, and IFNL1 were evaluated by RT-qPCR at 24 hpt, and mRNA levels were expressed as fold change (increases) in comparison to mock-treated cells, used as controls. Three different experiments were performed, with similar results. *P< 0.05, **P<0.01, ***P<0.001, ****P<0.0001 (using Student’s *t* test).

To test whether the negative effect of IFI6 on innate immunity also applies to cells from other origins, parental and IFI6 KO lung epithelial A549 cells were transfected with poly(I:C). As expected, after poly(I:C) treatment, high levels of IFIT2, IFNL1 (a type III IFN), and the proinflammatory cytokines CCL2, and CXCL10 were induced (**Figure 5A**). Interestingly, the levels of these genes were induced to higher levels in the IFI6 KO cells (using two different clones) compared to the parental cells (**Figure 5A**), further supporting that IFI6 modulates IFN and pro-inflammatory responses.

To assess the effect of IFI6 in conferring biologically relevant IFN-mediated antiviral activity to virus infection, we assessed the effect of knocking-out IFI6 on viral infection (**Figure 5B**). Control human A549 cells or IFI6 KO A549 (both clones) were transfected with poly(I:C) to induce an antiviral state and then, infected with rVSV-GFP (MOI 0.1). Then, rVSV-GFP production was analyzed at 24 hpi, as an indirect measure of the antiviral state induced by poly(I:C) on the cells. rVSV-GFP grew with high titers (~ 10^8^ pfu/ml) in mock-treated cells, irrespective of whether they were knocked-out or not for IFI6 (**Figure 5B**, left). In contrast, virus titers were decreased by more than 10,000-fold in poly(I:C)-transfected control cells, consistent with the induction of a host antiviral state in these cells (**Figure 5B**, right). Interestingly, in IFI6 KO cells transfected with poly(I:C), rVSV-GFP titers were 10-fold lower than titers in control cells (**Figure 5B**, right). These results correlate with our RT-qPCR data (**Figure 5A**) and further demonstrated that IFI6 expression decreases induction of host antiviral responses. Notably, we obtained similar results for both IFI6 KO A549 clones, further reinforcing the results.

To ascertain whether IFI6 modulates innate immunity after viral infections, A549 cells or A549 cells overexpressing hACE-2 were infected with IAV (MOI 3) and SARS-CoV-2 (MOI 1), respectively. The expression of IFIT2 and IFNL1 was measured by RT-qPCR at 24, and 48 hpi for IAV (**Figure 5C**) and SARS-CoV-2 (**Figure 5D**). As expected, IAV and SARS-CoV-2 infections induced the expression of IFIT2 and IFNL1 (**Figures 5C, D**). Interestingly, the levels of these genes were induced to higher levels in the IFI6 KO A549 cells compared to the parental A549 cells (**Figures 5C, D**).

**Figure 6:**
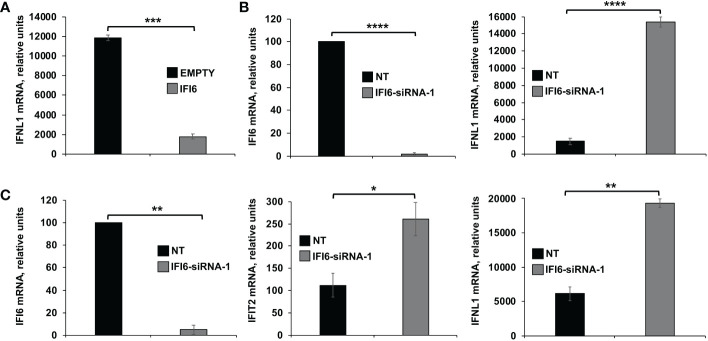
Effect of IFI6 on the modulation of innate immune responses induced in epithelial cells. *Effect of IFI6 overexpression.* **(A)** Human 293T cells were transfected with a plasmid expressing IFI6 (pCAGGS-IFI6-HA) or with the empty plasmid as control. *Effect of IFI6 silencing.* **(B, C)** Alternatively, 293T **(B)** and BEAS-2B **(C)** cells were transfected with the IFI6 siRNA or with the NT siRNA as control. **(A–C)** At 24 hpt, the cells were infected with SeV for an additional 24 h. The levels of IFI6 **(B, C)**, IFNL1 **(A–C)** and IFIT2 **(C)** expression were measured by RT-qPCR and mRNA levels were expressed as fold change (increases) in comparison to mock-treated cells, used as controls. Three different experiments were performed, with similar results. *P< 0.05, **P<0.01, ***P<0.001, ****P<0.0001 (using Student’s *t* test).

Additionally, since the above experiments were performed in knocked-down or knocked-out cells, overexpression experiments to further analyze the effect of IFI6 on innate immune responses were assessed. To this end, the highly-transfectable human 293T cells were transfected with a plasmid encoding IFI6 fused to an HA epitope tag, or with an empty plasmid as control. Then, cells were infected with SeV (MOI 3), to induce innate immune responses and the expression of IFNL1 was measured. As expected, SeV infection induced the expression of IFNL1 (12,000-fold). Remarkably, expression of IFNL1 was induced to a lower level (~6-fold less) in cells overexpressing IFI6 compared to control cells (**Figure 6A**). Conversely, and correlating with the overexpression data (**Figure 6A**), induction of IFNL1 in 293T cells infected with SeV was higher (~10-fold) when cells were previously knocked-down for IFI6 expression using an IFI6-specific siRNA (**Figure 6B**) than in control cells transfected with the NT siRNA (**Figure 6B**).

In addition, as all the previous cell lines used in the experiments are tumor-derived, experiments in the BEAS-2B cells, which is non-tumorigenic epithelial cell line derived from human bronchial epithelium, were performed (**Figure 6C**). Using this cell line, induction of IFIT2 and IFNL1 in cells infected with SeV was higher (2 to 3-fold) when cells were previously knocked-down for IFI6 expression using an IFI6-specific siRNA (**Figure 6C**) than in control cells transfected with the NT siRNA (**Figure 6C**). These results further confirmed that IFI6 counteracts IFN responses in different cell systems and using alternative approaches to induce innate immune responses.

### Effect of IFI6 on the induction of innate immune responses *in vivo*

**Figure 7:**
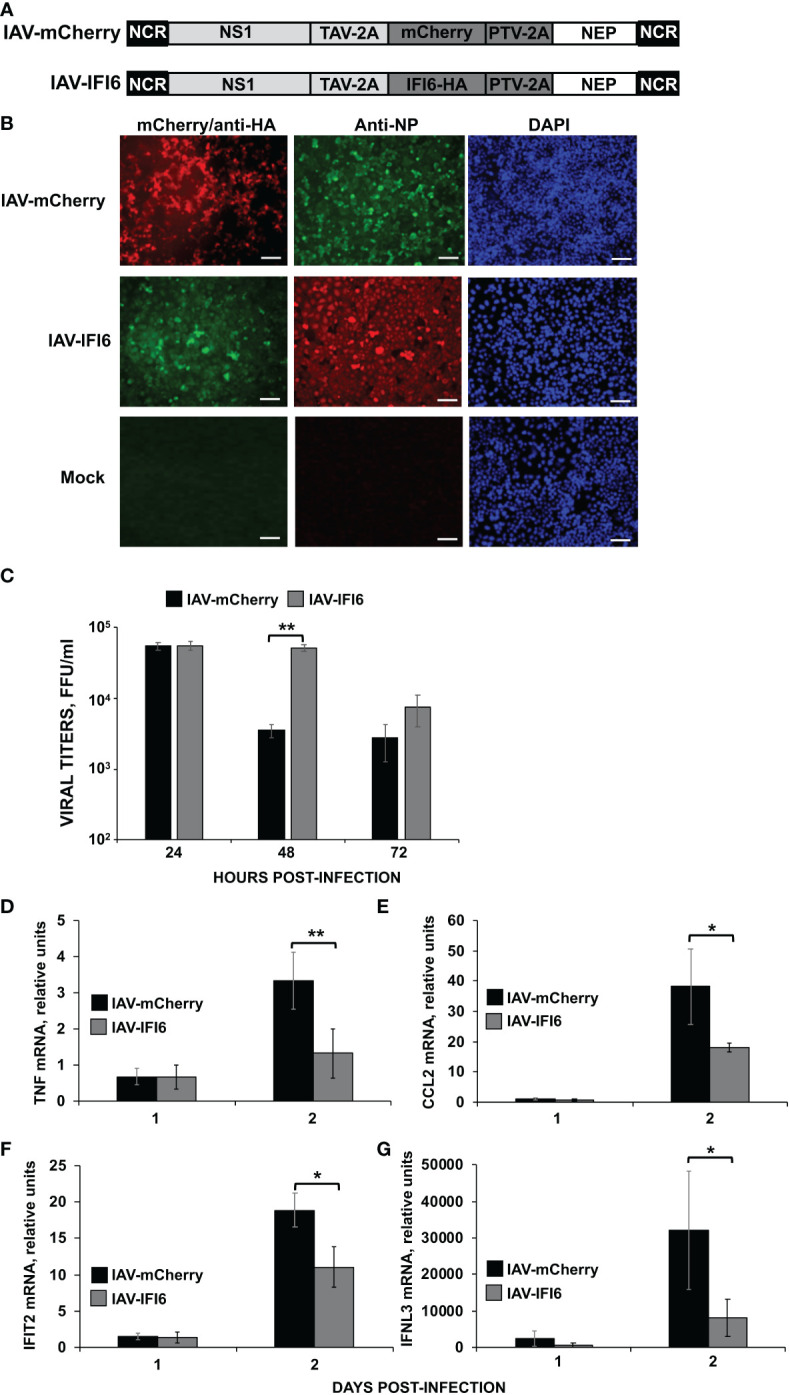
Effect of IFI6 expression on the induction of innate immune responses *in vivo*. **(A)** Schematic representation of PR8 viruses expressing mCherry and IFI6: A modified IAV-PR8 NS segment encoding NS1, mCherry (top) or IFI6-HA (bottom), and NEP are indicated. Black boxes at the beginning and end of each viral segment represent the viral 3’ and 5’ noncoding regions (NCR). White boxes indicate the viral NEP. Light gray boxes show the NS1 proteins. The thosea asigna virus (TAV) 2A 2A autoproteolytic cleavage site used for the expression of NS1 and mCherry/IFI6 and the porcine teschovirus (PTV) 2A autoproteolytic cleavage site used for the expression of mCherry/IFI6 and NEP are also indicated. **(B)** MDCK cells were non-infected (Mock) or infected (MOI 0.01) with IAV-mCherry and IAV-IFI6. At 24 hpi, cells were fixed and permeabilized and visualized for mCherry expression. Then, the cells were stained with anti-HA and anti-NP Abs. DAPI was used for nuclear staining. Representative images (20× magnification) are included. Scale bar, 50 μm. **(C)** A549 cells were infected with IAV-IFI6 and IAV-mCherry (MOI 0.1) and viral titers in the supernatants were measured by an immunofocus assay in MDCK cells. **(D–G)** Mice (n=4/group) were infected with IAV-mCherry and IAV-IFI6 viruses (2000 FFU/mice). At days 1 and 2 pi, TNF **(D)**, CCL2 **(E)**, IFIT2 **(F)**, and IFNL3 **(G)** expression was evaluated in mice lungs by RT-qPCR. Increases in mRNA levels were expressed as fold changes in comparison to mock-infected mice, used as controls. *P< 0.05, **P<0.01 (using Student’s *t* test).

We generated recombinant IAVs expressing IFI6 or mCherry (IAV-IFI6 and IAV-mCherry, respectively) to analyze the effect of IFI6 *in vivo*. To this end, IFI6 or mCherry ORFs were cloned in plasmids encoding an IAV NS split segment, so that the NS1, NEP, and IFI6 or mCherry proteins were flanked by the Thosea asigna virus (TAV) 2A autoproteolytic site (between NS1 and IFI6 or mCherry genes) and the porcine teschovirus (PTV) 2A autoproteolytic site (between IFI6 or mCherry and NEP genes) (**Figure 7A**). IAV-IFI6 and IAV-mCherry were generated using 8 ambisense plasmids as previously described. The sequence of the NS1/NEP segments encoding IFI6 and mCherry was confirmed by RT-PCR and Sanger sequencing. In addition, we confirmed that viruses express IFI6 and mCherry (**Figure 7B**). Interestingly, growth kinetics in A549 cells, showed that at 48 and 72 hpi, IAV-mCherry replicated to lower levels than IAV-IFI6 (**Figure 7C**), correlating with our results showing that IFI6 expression positively affects IAV replication (**Figures 2C, E**).

To analyze whether the overexpression of IFI6 affects viral replication and the induction of innate immune responses *in vivo*, mice were infected with the viruses (2,000 FFU/mice). Viral titers, as well as the induction of IFNL3, IFIT2 and pro-inflammatory cytokines (TNF and CCL2), were analyzed in mice lungs at 1 and 2 dpi. No significant differences in viral titers were observed at 1 and 2 dpi (**Supplementary Figure 1**). At 1 dpi, no induction of TNF, CCL2, and IFIT2 was observed in infected mice as compared to mock-infected mice (**Figures 7D–F**). The expression of IFNL3 increased to a limited degree at day 1 p.i., which interestingly, was slightly higher, although not statistically significant, in the mice infected with the IAV-mCherry, than in the mice infected with IAV-IFI6 (**Figure 7G**). In contrast, a clear induction of TNF, CCL2, IFIT2, and IFNL3 was observed in the infected mice lungs at 2 dpi. Interestingly, at 2 dpi, the induction of TNF, CCL2, IFIT2, and IFNL3 was higher in the lungs of mice infected with IAV-mCherry, than in the lungs of IAV-IFI6-infected mice (**Figures 7D–G**). These data indicate that this novel strategy may be a valid approach to analyze the effect of ISGs *in vivo*. In addition, these data correlate with data from **Figures 2**–**5** and further confirms that IFI6 negatively modulates innate immune responses *in vivo*.

### IFI6 binds to poly(I:C)

**Figure 8:**
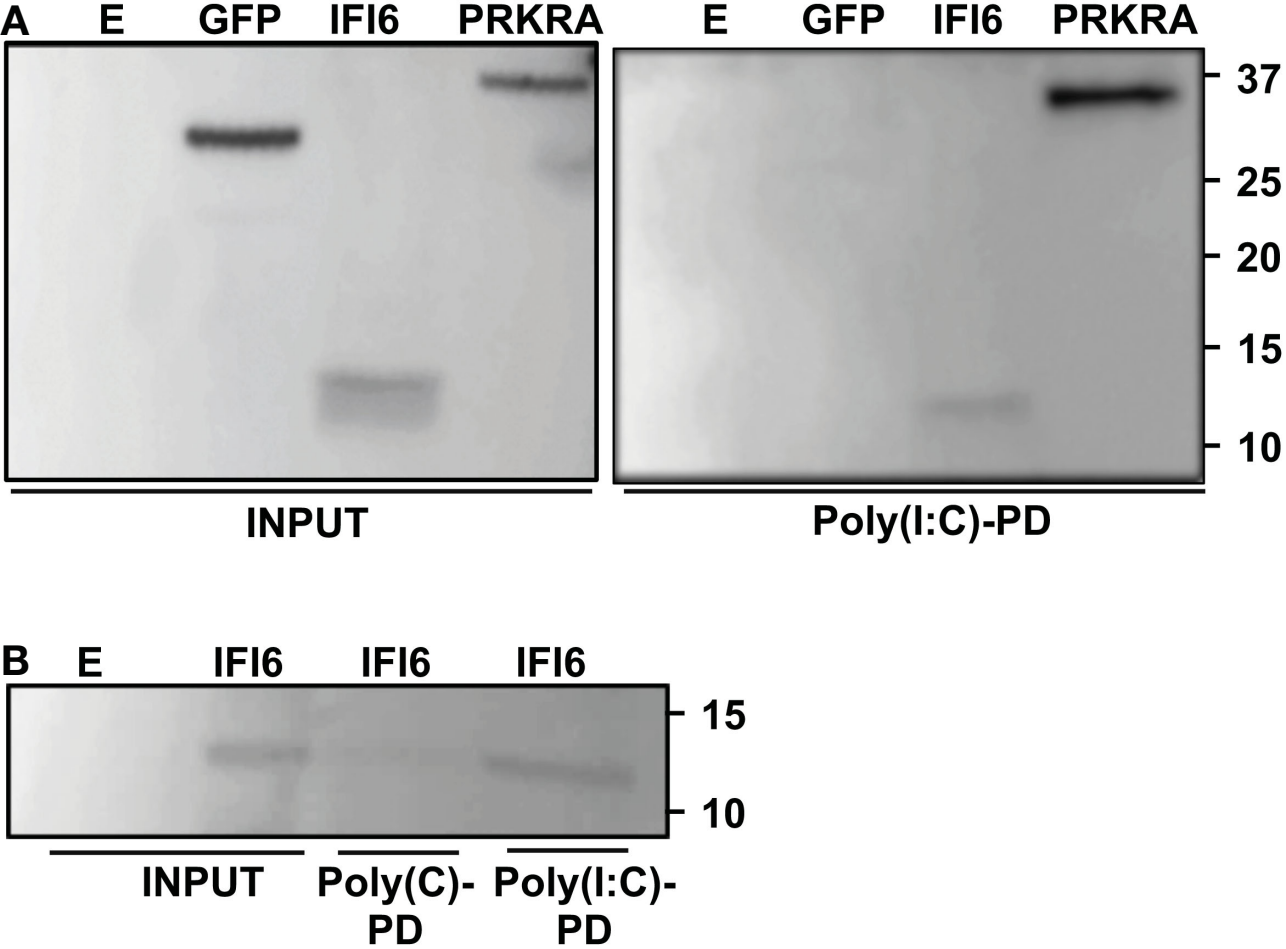
IFI6 binds poly(I:C). **(A)** Human 293T cells were transiently transfected with the pCAGGS plasmids encoding GFP, IFI6-HA and PRKRA-FLAG, or with an empty plasmid **(E)** Pull-down (PD) experiments using poly**(C)** (data not shown) and poly(I:C)-conjugated agarose beads were performed using cellular extracts. Western blotting using Abs specific for GFP, the HA tag (to detect IFI6) or the FLAG tag (to detect PRKRA) was performed to detect protein in the cellular lysates (Input) and after the pull-down (poly(I:C)-PD). Molecular weight markers are indicated (in kilodaltons) on the right. **(B)** Recombinant IFI6 protein fused to 6xHis and GST was incubated with poly**(C)** and poly(I:C)-conjugated agarose beads. A Western blot using an Ab specific for the GST tag (to detect IFI6) was performed to detect protein in the input and after the pull-down (polyC-PD and poly(I:C)-PD). Two independent experiments and Western blots were performed, with similar results.

Bioinformatic predictions using the RNABindRplus method, that combines sequence homology-based methods and machine learning for improving the reliability of predicted RNA-binding residues in proteins (40, 45), showed that IFI6 encodes 15 residues which could putatively bind RNA. To experimentally analyze whether IFI6 binds RNA, and in order to explain its role as a negative modulator of innate immune responses, 293T cells were transfected with an HA-tagged IFI6 expressing plasmid. GFP and PRKRA, a known dsRNA-binding protein (46), expressing plasmids were used as negative and positive controls, respectively. Then, cellular lysates were bound to agarose beads conjugated with poly(I:C), an analog of dsRNA, or poly(C) as control. Interestingly, IFI6 was pulled-down using poly(I:C)-conjugated agarose beads (**Figure 8A**) but not with the poly(C)-conjugated agarose beads (data not shown). As expected, GFP was not pulled-down using neither poly(I:C) nor poly(C) agarose beads; and PRKRA was detected using poly(I:C)-conjugated agarose beads but did not bound to agarose beads conjugated to poly(C) (**Figure 8A**), strongly suggesting that IFI6 protein binds poly(I:C).

To further confirm the binding of IFI6 to poly(I:C), and to rule out that the binding could be mediated by other proteins present in the cellular extracts, an IFI6 recombinant protein fused to GST and 6xHis tags, was expressed in *E. coli* and purified with an Ni-column. Purified recombinant IFI6 was incubated with poly(C) or poly(I:C)-conjugated agarose beads. As expected, recombinant IFI6 bound to poly(I:C)-agarose beads but not to poly(C)-agarose beads (**Figure 8B**), further confirming that IFI6 binds poly(I:C), and suggesting that the binding of IFI6 to poly(I:C) is not mediated by other cellular proteins.

### IFI6 interacts with RIG-I

**Figure 9:**
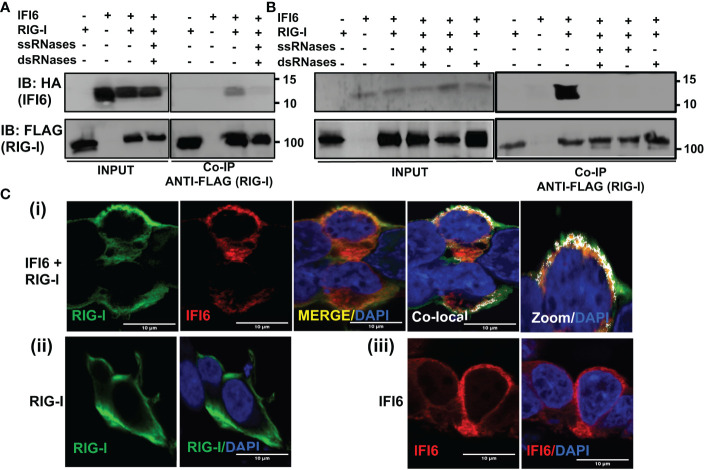
IFI6 interacts with RIG-I. **(A, B)** Human 293T cells were transiently co-transfected with the pCAGGS plasmids encoding IFI6-HA and RIG-I-FLAG, or with empty plasmids. Then, the cells were transfected with poly(I:C) during 24 h **(A)** or infected with SeV for 24 h **(B)**. **(A, B)** Cellular extracts were either treated with RNases digesting ssRNAs (ssRNases), RNases digesting dsRNAs (dsRNases), or non-treated with RNases. Coimmunoprecipitation (Co-IP) experiments using anti-FLAG to pull down RIG-I using affinity columns were performed. Western blotting using antibodies specific for the HA tag (to detect IFI6, top panels) or the FLAG tag (to detect RIG-I protein, bottom panels) was performed to detect protein in the cellular lysates (Input) and after the Co-IP. IB, immunoblot. Molecular weight markers are indicated (in kilodaltons) on the right. Western blots after the IP were quantified by densitometry using ImageJ software, and the amounts of IFI6 were normalized to the amounts of RIG-I (numbers below the top blot). ND, not detected. Two independent experiments and Western blots were performed, with similar results. **(C)** Human A549 cells were transiently co-transfected with the pCAGGS plasmids encoding IFI6-HA and RIG-I-FLAG, or with empty plasmids. Then, the cells were transfected with poly(I:C) during 24 h. At 24 hpt, cells were fixed with paraformaldehyde, and RIG-I-FLAG and IFI6-HA were labeled with antibodies specific for the tags (in green and red, respectively), and nuclei were stained with DAPI (in blue). Pictures show cells co-transfected with plasmids expressing RIG-I and IFI6 together (i), and cells transfected with the plasmids expressing RIG-I (ii) and IFI6 (iii) separately. (i) Areas of co-localization of both proteins appear in yellow in the third picture and in white in the fourth picture. A zoom of the colocalization image (from fourth picture) is depicted in the fifth picture. Scale bar, 10 μm.

SARS-CoV-2 (47–49), IAV (50, 51), SeV (51), and transfected poly(I:C) (52) get sensed inside the cells through RIG-I. Taking into account that we observe a negative effect of IFI6 on innate immune responses induced after SARS-CoV-2, IAV and SeV infections, and after poly(I:C) transfection, and that IFI6 binds poly(I:C) and possibly RNAs, as RIG-I, we hypothesized that IFI6 could be modulating RIG-I activation through binding to RIG-I. To test this hypothesis, cells were transfected with plasmids expressing RIG-I fused to a FLAG epitope tag and IFI6 fused to an HA epitope tag, and then transfected with poly(I:C). Cellular extracts were subjected to immunoprecipitation using anti-FLAG affinity columns. Remarkably, RIG-I and IFI6 co-immunoprecipitated together, suggesting that these two proteins directly, or indirectly, interact (**Figure 9A**). To determine whether the interaction of IFI6 and RIG-I is mediated by RNAs, an immunoprecipitation was performed under the same experimental conditions but in the presence of RNAseA and RNaseT1 to digest ssRNAs, and with RNAseIII to digest dsRNAs, which could be present in the cellular extracts (**Figure 9A**). In this case, the amount of IFI6 protein co-immunoprecipitated with RIG-I was clearly decreased, almost to undetectable levels, whereas the amount of IFI6 protein in the cellular extracts before the immunoprecipitation was very similar in the extracts without treatment and with RNAses-treatment (**Figure 9A**). These results strongly suggested the interaction of IFI6 and RIG-I is mediated, at least in part, by RNAs.

To further assess the RNA-mediated interaction of IFI6 with RIG-I in a more physiological system, similar experiments were performed using SeV-infected cells (**Figure 9B**). Again, IFI6 interacted with RIG-I in SeV-infected cells (**Figure 9B**). Interestingly, the interaction was clearly decreased when the cell extracts were previously treated with RNAseA and RNaseT1 to digest ssRNAs, with RNAseIII to digest dsRNAs, or with the three RNAses together (**Figure 9B**). These results strongly suggested an RNA-mediated interaction of IFI6 with RIG-I after SeV infection.

To study a likely intracellular colocalization of IFI6 and RIG-I, cells were transiently transfected with pCAGGS plasmids expressing RIG-I fused to a FLAG epitope tag, and IFI6 fused to an HA epitope tag, the cells were transfected with poly(I:C), and the subcellular localization of both proteins was determined by immunofluorescence and confocal microscopy (**Figure 9C**). IFI6 and RIG-I expression was detected in the cytoplasm, showing a similar pattern when IFI6 and RIG-I were expressed together or separately (**Figure 9C**). Importantly, a partial co-localization of IFI6 and RIG-I was observed in distal regions of the cytoplasm (**Figure 9C**), reinforcing the co-IP experiments and further demonstrating that IFI6 and RIG-I can interact inside the cell (**Figures 9A, B**).

### IFI6 affects modulation of innate immune responses mediated by RIG-I

**Figure 10:**
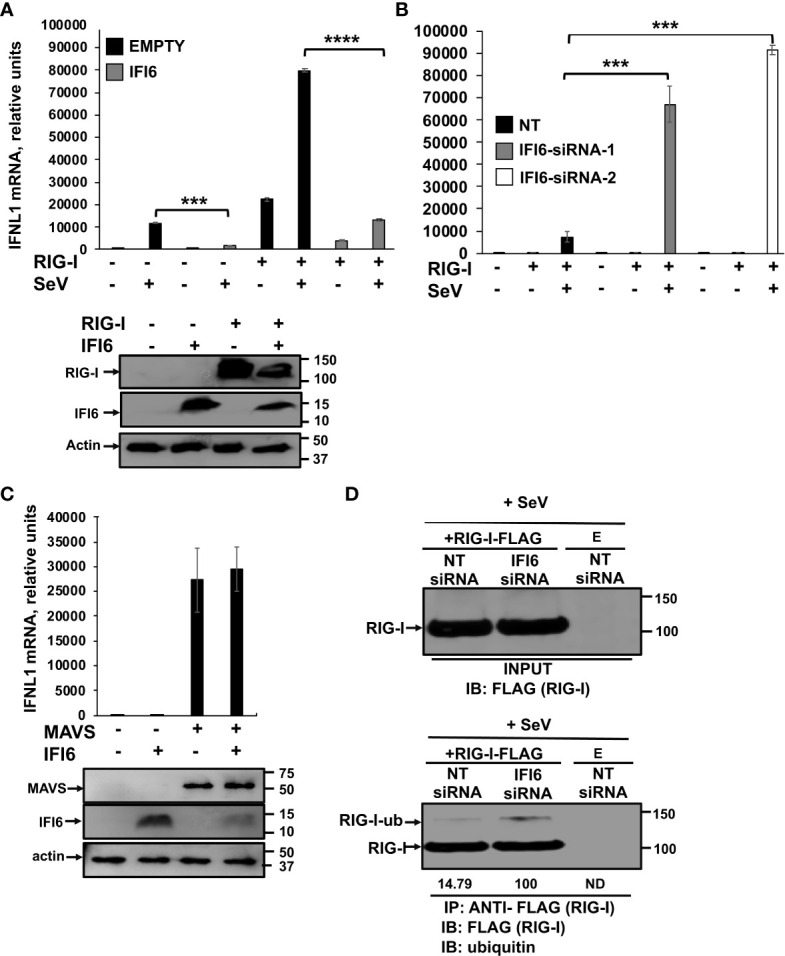
IFI6 affects RIG-I activation. **(A)** Human 293T cells were transiently co-transfected with the pCAGGS plasmids encoding IFI6-HA and RIG-I-FLAG, or with empty plasmids **(E)**. Then, the cells were infected with SeV (MOI 3) during 24 h. Proteins in the cellular extracts from non-infected cells (bottom blots in **A**) were subjected to Western blotting using antibodies specific for FLAG tag (to detect RIG-I protein), the HA tag (to detect IFI6) or actin, as loading control. **(B, D)** Human 293T cells were transfected with two **(B)** or one **(D)** siRNAs specific for IFI6 or with the NT siRNA and 24 h after siRNA-transfections, the cells were transfected with the pCAGGS plasmid encoding RIG-I-FLAG, or with the empty plasmid, as control. Then, the cells were infected with SeV (MOI 3) during 24 h. **(C)** Human 293T cells were transiently co-transfected with the pCAGGS plasmids encoding IFI6-HA and MAVS-FLAG, or with empty plasmids, during 24 h. Proteins in the cellular extracts from non-infected cells (bottom blots in **C**) were subjected to Western blotting using antibodies specific for the FLAG tag (to detect the MAVS protein), the HA tag (to detect IFI6) or actin, as loading control. **(A–C)** Total RNAs were extracted and the levels of IFNL1 were measured by RT-qPCR and mRNA levels were expressed as fold change (increases) in comparison to control cells, used as controls. Three different experiments were performed, with similar results. ***P<0.001, ****P<0.0001 (using Student’s *t* test). **(D)** Cellular extracts (input) were subjected to immunoprecipitation (IP) using an anti-FLAG antibody, to pull down RIG-I using affinity columns. Proteins in the cellular extracts and after IP were subjected to Western blotting using antibodies specific for ubiquitin or the FLAG tag (to detect RIG-I protein). IB, immunoblot. Western blots after the IP were quantified by densitometry using ImageJ software, and the amounts of RIG-I ubiquitinated (RIG-I-ub) were normalized to the amounts of RIG-I (numbers below the blot). ND, not detected. Two independent experiments and Western blots were performed, with similar results.

To further analyze whether IFI6 negatively modulates the induction of innate immune responses mediated by RIG-I, 293T cells were transiently transfected with the plasmid expressing RIG-I-FLAG in the presence and absence of the plasmid expressing IFI6-HA. Then, the cells were either mock-infected or infected with SeV (**Figure 10A**). The expression levels of RIG-I, and IFI6 were confirmed by Western blot using antibodies specific for FLAG (to detect RIG-I), and HA (to detect IFI6) (**Figure 10A**). According to previous results (53), overexpression of RIG-I induced the expression of IFNL1, that was further induced in the SeV-infected cells (~80,000-fold, **Figure 10A**). Remarkably, overexpression of IFI6 led to decreased IFNL1 induction in RIG-I overexpressing cells, mock- (~3,000-fold induction) or SeV-infected (~14,000-fold induction, **Figure 10A**). To confirm these results, 293T cells silenced for IFI6 expression using two different siRNAs and transfected with the plasmid expressing RIG-I-FLAG were either mock- or SeV-infected (**Figure 10B**). The highest levels of IFNL1 induction were observed in cells overexpressing RIG-I and infected with SeV, as expected. Furthermore, in this case, IFNL1 induction was higher in cells silenced for IFI6 expression as compared to control transfected cells (**Figure 10B**), further supporting that IFI6 modulates RIG-I activation. In contrast to the effect of IFI6 on RIG-I activation, no effect of IFI6 overexpression was observed when the levels of IFNL1 were induced after the overexpression of MAVS, an scaffold adaptor involved in RIG-I activation (54) (**Figure 10C**). The expression levels of MAVS, and IFI6 were confirmed by Western blot using antibodies specific for FLAG (to detect MAVS), and HA (to detect IFI6) (**Figure 10C**). These data suggest that the effect of IFI6 in negatively modulating RIG-I activation is specific, as it does not affect MAVS activation.

Since polyubiquitination is essential for RIG-I activation (55–57), we next analyze whether binding of IFI6 to RIG-I affects RIG-I activation, therefore, leading to the negative modulation of innate immune responses, by affecting levels of RIG-I ubiquitination. To this end, expression of IFI6 was knocked-down in 293T cells using siRNA and then cells were transfected with a plasmid expressing RIG-I-FLAG, and infected with SeV to induce RIG-I activation. RIG-I was immunoprecipitated using and anti-FLAG Ab (**Figure 10D**) and the levels of ubiquitinated RIG-I were determined by Western blot. As expected, RIG-I was ubiquitinated to higher levels in cells knocked-down for IFI6 than in the control cells (**Figure 10D**), suggesting that IFI6 negatively affects RIG-I activation by preventing its activation, an effect likely mediated by the interaction of IFI6 with RIG-I.

## Discussion

In this study, we identified an uncovered, novel function of IFI6 in negatively regulating innate immune responses induced after infection with viruses belonging to different families (i.e. IAV, SARS-CoV-2, and SeV), as well as after poly(I:C) transfection, even in mice, using a novel IAV system to overexpress IFI6 in infected cells. In addition, we find that silencing or knocking-out the expression of IFI6 reduces the production of IAV and SARS-CoV-2 infectious viruses, likely due to the effect of IFI6 on modulating antiviral responses.

IFI6 is an ISG whose expression is induced by many viruses (15, 17, 38, 39), such as IAV (**Figure 1C**), and SARS-CoV-2 (**Figure 1D**). Many ISGs display antiviral functions (1). However, as an excessive antiviral signaling can be detrimental to the host, many host factors play a role in negatively modulating innate immune responses. In this sense, we previously described that the ISGs IFI44 and IFI44L display this feedback regulatory functions (10, 11), and others have described that other ISGs, such as IFI35 and ISG56/IFIT1 negatively regulate antiviral responses (58, 59). Correlating with the effect of IFI6 in negatively modulating IFN responses, we observed decreased IAV and SARS-CoV-2 titers in cells silenced or knocked-out for IFI6 expression, compared to the control cells (**Figures 2C, E, F**). Furthermore, decreased rVSV-GFP titers in poly(I:C)-transfected cells silenced or knocked-out for IFI6 compared to poly(I:C)-transfected control cells were observed (**Figures 4B, F**, and **5B**). Similarly, silencing of IFI35 and ISG56/IFIT1, proteins which negatively modulate IFN responses, decrease VSV replication (58, 59), and silencing of IFI44 and IFI44L decrease IAV and coronavirus replication (10, 11). DDX60 overexpression, a gene upregulated after viral infections, which has been shown to bind RIG-I, increasing RIG-I activation, decreased VSV and poliovirus replication (60). Similarly, DHX15, a gene upregulated after viral infections, which has been shown to bind RIG-I, increasing RIG-I activation, increased encephalomyocarditis virus (EMCV) replication (61), and the helicases DHX16 and DDX6, two proteins which recognize specific viral RNA to trigger RIG-I-dependent innate antiviral immunity negatively affect IAV, Zika and SARS-CoV-2 (62) and enterovirus (63) replication.

Intriguingly, in our experiments in mice infected with IAVs overexpressing IFI6 and mCherry, we do not observe a significant difference in viral titers in mouse lungs between both viruses (**Supplementary Figure 1**), although we do observe a difference in A549 cells (**Figure 7C**). Since we observe upregulation in IFN responses, as determined by measuring the expression of IFIT2 and IFNL3 by RT-qPCR, in IAV-mCherry-infected mice compared to IAV-IFI6-infected mice (**Figures 7F, G**), this could be due to the fact that the differences in innate immune responses are not high enough to significantly affect viral replication, or to the fact that IFI6 or mCherry expression is modulating viral replication by other mechanisms distinct from IFN responses. In any case, future directions will include the testing of IFI6 functions using IFI6 KO mice, however, as far as we are concerned, these mice are not available yet.

We show for the first time that IFI6 binds poly(I:C), an analog of dsRNA (**Figure 8**). In addition, we show that IFI6 binds RIG-I, in poly(I:C)-transfected and SeV-infected cells (**Figures 9A, B**, respectively) and that IFI6 and RIG-I partially colocalize intracellularly (**Figure 9C**), supporting an interaction between these two proteins. Furthermore, we found that the binding of IFI6 to RIG-I is most likely mediated by RNA, since treatment of poly(I:C)-transfected cell extracts and SeV-infected cell extracts with RNAses, decreases the binding of IFI6 to RIG-I (**Figures 9A**, **3B**). This data also supports that IFI6 binds SeV viral RNAs, and therefore, more likely, other viral RNAs. Upon viral recognition, RIG-I interacts with MAVS (also known as cardif, IPS-1 and VISA), an interaction which favors the activation of IRF-3, IRF-7 and NF-κB (64, 65). This process triggers the expression of multiple proinflammatory factors and antiviral genes, such as IFN and ISGs, which inhibit viral replication (64, 65). Interestingly, IFI6 is a protein localized to the mitochondria (16), like RIG-I and MAVS, reinforcing our data showing that IFI6 and RIG-I partially colocalize (**Figure 9C**) and interact (**Figures 9A, B**) inside the cell.

RIG-I is a PRR that recognizes different viral infections, such as the ones we have used in this work [e.g. SARS-CoV-2 (47, 48, 66), IAV (50, 51), and SeV (51)], as well as transfected poly(I:C) (52), being its activation tightly regulated [reviewed in (13)]. RIG-I endogenously exists in the cytoplasm of the cell in a phosphorylated and inactivated conformation when it is not activated by RNAs. Phosphorylation is mediated at the N-terminal CARD domains of RIG-I by PKC-α/β and at the CTD domain by CKβ (13). RIG-I gets acetylated at K909 in its C terminal domain, requiring deacetylation by HDAC6 to be able to recognize RNA in its activated form (13). Upon recognition of dsRNA, RIG-I unfolds into an open and activated state that is mediated by the flexible hinge regions between the CARD domains and the helicase domain, and between the helicase and the C terminal domain. Then, RIG-I activation involves the ubiquitination of the protein by the E3 ubiquitin ligases Riplet and TRIM25, and de-phosphorylation of the CARD domain by the phosphatase PP1-α/γ (13). In this sense, cellular proteins, such as CYLD, USP3, USP14, USP21, and USP27X suppress antiviral immune responses by deubiquitinating RIG-I (67–71). Furthermore, other host proteins, such as the helicases DDX6, DHX15, DHX29, and DDX60 have been identified as RIG-I cofactors that interact with RIG-I and with viral RNAs and dsRNAs, thereby increasing RIG-I activity (60, 61, 72, 73). However, unlike the interaction of IFI6 with RIG-I, which seems to be mediated by RNAs, the interaction of DHX29 and DDX60 with RIG-I seems independent of RNAs (60, 72). Furthermore, IFI16 also interact with RIG-I, but in this case the interaction increase RIG-I activity after IAV infections (74).

In the cell, in addition to RIG-I, there are other sensors detecting viral RNAs, which initiate IFN responses, such as MDA-5 and TLR-3 (3, 13). Thus, whether IFI6 affects the activation of these other receptors, and therefore, the induction of innate immune responses mediated by these other PRRs is a possibility that needs further investigation.

In summary, we have described a completely undiscovered function for IFI6 in impairing host IFN and inflammatory responses. In addition, the results show that blocking IFI6 expression by either silencing or knocking-out IFI6 decreases virus production. Importantly, these new functions for IFI6 may be targeted for treating viral-mediated diseases and also for controlling diseases associated to hyperinflammation.

## Data availability statement

The datasets presented in this study can be found in online repositories. The names of the repository/repositories and accession number(s) can be found below: https://www.ncbi.nlm.nih.gov/bioproject/PRJNA916250, PRJNA916250 (SRA).

## Ethics statement

The animal study was reviewed and approved by Procedures involving animals were approved by the CSIC ethics committee for animal experimentation and by the Division of Animal Protection of the regional government of Madrid in compliance with national and European Union legislation (PROEX89.5/20).

## Author contributions

LV, VR and DL-G: Investigation, Methodology, Validation, Formal analysis, Data curation, Writing – Review & Editing. DT: Conceptualization, Resources, Writing – Review & Editing. LM-S: Conceptualization, Resources, Writing – Review & Editing. AN: Conceptualization, Investigation, Methodology, Validation, Formal analysis, Data curation, Writing – Review & Editing. MD: Conceptualization, Resources, Investigation, Methodology, Validation, Formal analysis, Data curation, Writing – Original draft, Writing – Review & Editing, Visualization, Supervision, Funding acquisition. All authors contributed to the article and approved the submitted version.

## Supplementary material

Supplementary Figure 1 Effect of IFI6 expression on viral replication *in vivo*. Mice (n=4/group) were infected with IAV-mCherry and IAV-IFI6 viruses (2,000 FFU/mice). At 1 and 2 dpi, viral titers in mouse lungs were evaluated by an immunofocus assay in MDCK cells.
