# Supplementary figures and images for: Interferon alpha inducible protein 6 is a negative regulator of innate immune responses by modulating RIG-I activation
Source: Front Immunol. 2023 Jan 30;14:1105309. doi: 10.3389/fimmu.2023.1105309 (PMC9923010; doi:10.3389/fimmu.2023.1105309)

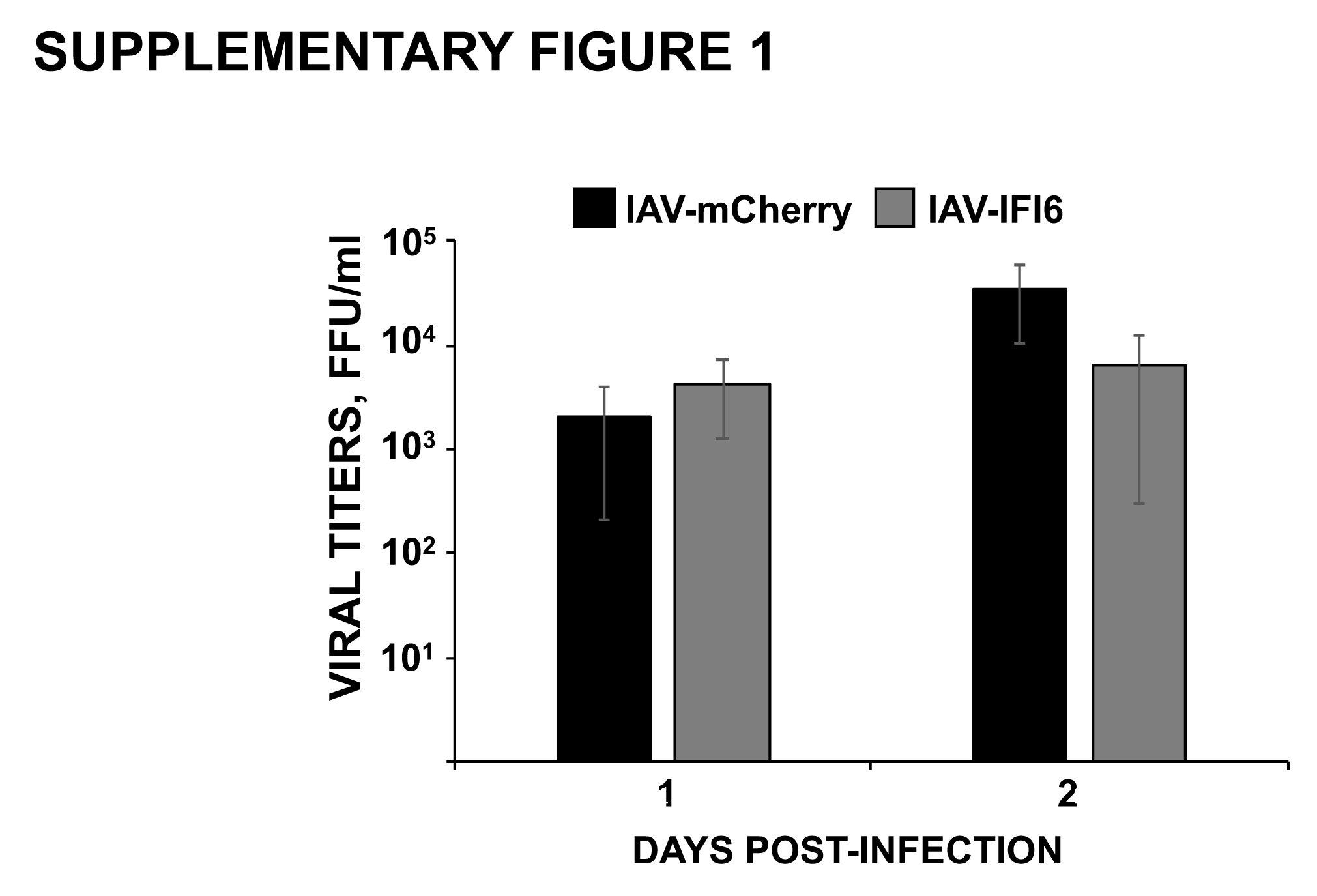

Supplement: Supplementary Figure 1 — Effect of IFI6 expression on viral replication in vivo. Mice (n=4/group) were infected with IAV-mCherry and IAV-IFI6 viruses (2,000 FFU/mice). At 1 and 2 dpi, viral titers in mouse lungs were evaluated by an immunofocus assay in MDCK cells. [file Image_1.tif]
